# Data based predictive models for odor perception

**DOI:** 10.1038/s41598-020-73978-1

**Published:** 2020-10-13

**Authors:** Rinu Chacko, Deepak Jain, Manasi Patwardhan, Abhishek Puri, Shirish Karande, Beena Rai

**Affiliations:** Tata Research Development and Design Centre, Tata Consultancy Services, 54-B, Hadapsar Industrial Estate, Pune, 411013 India

**Keywords:** Cheminformatics, Computer science, Information technology, Scientific data, Statistics

## Abstract

Machine learning and data analytics are being increasingly used for quantitative structure property relation (QSPR) applications in the chemical domain where the traditional Edisonian approach towards knowledge-discovery have not been fruitful. The perception of odorant stimuli is one such application as olfaction is the least understood among all the other senses. In this study, we employ machine learning based algorithms and data analytics to address the efficacy of using a data-driven approach to predict the perceptual attributes of an odorant namely the odorant characters (OC) of “sweet” and “musky”. We first analyze a psychophysical dataset containing perceptual ratings of 55 subjects to reveal patterns in the ratings given by subjects. We then use the data to train several machine learning algorithms such as random forest, gradient boosting and support vector machine for prediction of the odor characters and report the structural features correlating well with the odor characters based on the optimal model. Furthermore, we analyze the impact of the data quality on the performance of the models by comparing the semantic descriptors generally associated with a given odorant to its perception by majority of the subjects. The study presents a methodology for developing models for odor perception and provides insights on the perception of odorants by untrained human subjects and the effect of the inherent bias in the perception data on the model performance. The models and methodology developed here could be used for predicting odor characters of new odorants.

## Introduction

Smells are ubiquitous in our daily life, ranging from the sweet pleasing aroma of a flower to the putrid smell emanating from a garbage dump. Historically, perfumes were luxuries enjoyed primarily by the royalty. However, with the advent of synthetic chemistry, fragrances are no longer an indulgence privy only to the aristocrats or the elites of the society^1,2^. Perfumes, deodorants and body sprays are now essential day-to-day personal care products and play a significant role in personal grooming. Fragrances are also widely added to various chemical products or formulations (body lotions, soaps, creams, detergents etc.) in order to improve their sensorial attributes by masking the otherwise “chemical” smell of the products^3^. Manufacturers frequently brief the fragrance houses about the changing social trends of consumer preferences, in anticipation that the fed information would lead to development of new products or variations in existing formulations^4^. The fragrance houses have experts trained in the art of making perfumes with knowledge on a variety of aromatic ingredients and their smells. The perfumer is tasked with creating a blend of ingredients that epitomizes the brief from the manufacturer.

Olfaction is the least understood among all senses, in terms of what causes an odorant to smell the way it does. Research indicates that there may be differences in the perception of smells by experts and untrained subjects^5^. Much of the ambiguity in the sense of smell comes from the subjective nature of odor perception^6–8^. There is little agreement in the industry regarding the measurement of odorant properties such as quality, intensity and similarity. Researchers collect odor perception data through a variety of approaches such as verbal profiling, similarity ratings and sorting^9,10^. In verbal profiling, subjects are asked to rate the odors against a set of predefined semantic descriptors that they would associate with the odors^11,12^. Such an approach requires an individual to make a comparison between an actual sensation and an abstract sensation based on their interpretation of the semantic descriptors. The definition of these semantic descriptors and the odor quality perception could be impacted by cultural differences, age, gender and several other demographic variables^5,13,14^. It has also been reported that with training or through experiences accumulated over time, the discrimination of odor quality can be improved^15^.

In order to come up with novel fragrances, a synthetic chemist needs to understand the relationship between odor quality and the physico-chemical properties of the odorant. A molecule can be called an odorant if it satisfies the following conditions. Firstly, the molecule must be sufficiently volatile in order to be transported through air to the nasal cavity so as to enable the molecule to interact with the olfactory receptors. Therefore, the molecule needs to be of a low molecular weight, typically less than 500 Da with a certain degree of hydrophobicity. Secondly, it must be an agonist of a receptor so that the chemical message carried by the molecule can be converted to a neuronal influx which can then be decoded by the olfactory bulb in the brain^16^. Researchers over the years have tried to unravel the relationship between the physical stimulus and the olfactory perception^17–24^. However, the stimulus-percept problem is riddled with several challenges. The size and dimensionality of the olfactory perceptual space is unknown in addition to the unpredictability of structure-odor relationships^25–27^. Structurally similar molecules can show very distinct odor profiles while molecules with diverse structures can show similar odors. Typical examples include enantiomers such as S-limonene which exudes a lemony smell whereas R-limonene has an orange odor^28^. Isovanillin despite its structural similarity to vanillin does not have the characteristic vanilla smell associated with vanillin^29^. On the other hand, cyclooctane and hexachloroethane are examples of molecules having a camphoraceous odor despite their very diverse structures^21^. The odor characteristics can also vary with the concentration of the odorant in the environment further complicating the rationalization of odor perception. For example, indole has a floral smell at low concentrations while it smells putrid at higher concentrations^30^.

It has been argued that experiential factors such as memory are critical for odor discrimination as opposed to just the chemical features of the stimulus^31^. Nevertheless, with developments in the field of machine learning, recent years have seen a growing interest in using a data-driven approach for prediction of structure-odor relationships. Many have attempted to demonstrate the feasibility of predicting olfactory perception with structural parameters of the odorant molecules as features^32–35^. However, the approaches used differ greatly with respect to the data used for the predictions, where some utilize perceptual data obtained from untrained individuals while others use qualitative data from trained experts thus making comparison difficult. Dravnieks and coworkers created a database of 160 odorants with 146 descriptors or words in order to describe the odor quality^36^. Licon et al. recently developed a database of 1689 odorants and a subgroup discovery algorithm based computational method to discover rules linking chemistry with perception. They analyzed 11 olfactory qualities namely sulfuraceous, vanillin, phenolic, musk, sandalwood, almond, orange-blossom, jasmine, hay, tarry and smoky. Musky odors were reported to be heavy molecules with large surface area and hydrophobicity value^37^. Shang et al. presented a proof-of-concept study of plausible replacement of a human panelist in Gas chromatography/Olfactometry (GC/O). They developed classification models for 10 odor descriptors using a database of 1026 odorants and reported 97% accuracy through a support vector machine model combined with feature extraction using boruta algorithm. They used oversampling technique to account for imbalance in the dataset; however, the oversampling step was performed before the splitting of the train and test data. This caused the model to see the test set compounds during the training itself thereby, overestimating their results^33^. Keller et al. organized the crowd-sourced DREAM Olfaction Prediction Challenge to develop machine learning algorithms for accurate prediction of the sensory attributes such as odor intensity, pleasantness and eight semantic descriptors of 480 structurally diverse odorant molecules. The random forest algorithm was used to train 1089 regression models to predict perceptual ratings of the subjects using 4884 molecular parameters. The correlation between the observed and predicted ratings was used as the performance metric^32,38,39^. They attempted to relate the chemical features to the perceptual ratings, however the discussion was restricted to the “decayed” odor character alone and the large number of features used in the model development does not allow one to understand the chemical information carried by these features. Additionally, the authors did not consider dilution as a feature for the models and only used the average of the ratings at the two dilutions to increase the number of samples for model training. Zhang et al. established Convolutional Neural Network (CNN) based predictive models for odor character and odor pleasantness to develop a molecular design/screening methodology for fragrance molecules with each molecule represented by a single odor character. They reported that the model correctly classified the odor characters of three out of four sweet odorants that were not part of the training data, however odorants exhibiting other odor characters were not tested^34^. The authors used all the 480 molecules as the training data and did not provide any information on model-tuning or its performance on a validation set. Therefore, one cannot infer if the deep learning model was overfitting to the training data. Furthermore, by testing the model on molecules of one odor character class alone, they failed to establish if the model had acceptable performance on the other classes as well.

In this study, we aim to use the perception data of untrained subjects and employ a machine learning-based classification approach for prediction of the “sweet” and “musky” odor characters (OC) of odorant molecules using 196 two-dimensional structural parameters and dilution as input features representing the odorants. Here, we annotate each molecule by the top three odor characters most associated with it, by the majority of the subjects of the psychophysical study by Keller et al.^38^ This takes into consideration the multiple odor qualities that could be exhibited by an odorant. This also reduces the impact of the differences arising due to subjects’ attributes such as gender and culture by focusing on the population behaviour alone. Various machine learning algorithms namely support vector machines, random forest, gradient boosting machine, adaptive boosting, extreme gradient boosting and k-nearest neighbors are compared to develop the optimal models for classification of the “sweet” and “musky” odor characters. Additionally, we analyze the misclassified molecules and employ data visualization techniques to comment on the efficacy of using psychophysical datasets for prediction of odor character. The models trained using extreme gradient boosting algorithm, were found to be the optimal models for both the classification tasks.

## Results

### Description of the data

As discussed earlier, odorants can be characterized based on several qualitative descriptors including their hedonic attributes. A variety of olfaction data with semantic description of an odorant molecule are available, however the disparity in the semantics leads to a vast number of labels for the prediction tasks. Therefore, in this study, the psychophysical dataset developed by Keller et al.^38^ was utilized to develop machine learning-based classification models for prediction of odor characters. The dataset consists of odorant property data for 480 structurally diverse compounds at two dilutions (concentrations). Approximately 13.3% samples were evaluated at the concentration of 1/10, 43.4% at 1/1000, 37.1% at 1/100,000 and 6.2% at 1/10,000,000. The odor properties included were odor familiarity, intensity, pleasantness and 20 semantic descriptors relating to odor quality. The semantic descriptors were acid, ammonia/urinous, bakery, burnt, cold, chemical, decayed, edible, fish, flower, fruit, garlic, grass, musky, sour, spices, sweaty, sweet, warm and wood. Each of these perceptual attributes were rated by 55 subjects in the range 0 to 100 based on how well each of the semantic descriptors applied to the odor. Each of the 55 subjects gave ratings to 1000 odor stimulus. During the original study, the subjects profiled 100 stimuli during each of their ten visits and carried out the study at a typical pace of one stimulus per minute.

### Data visualization

**Figure 1:**
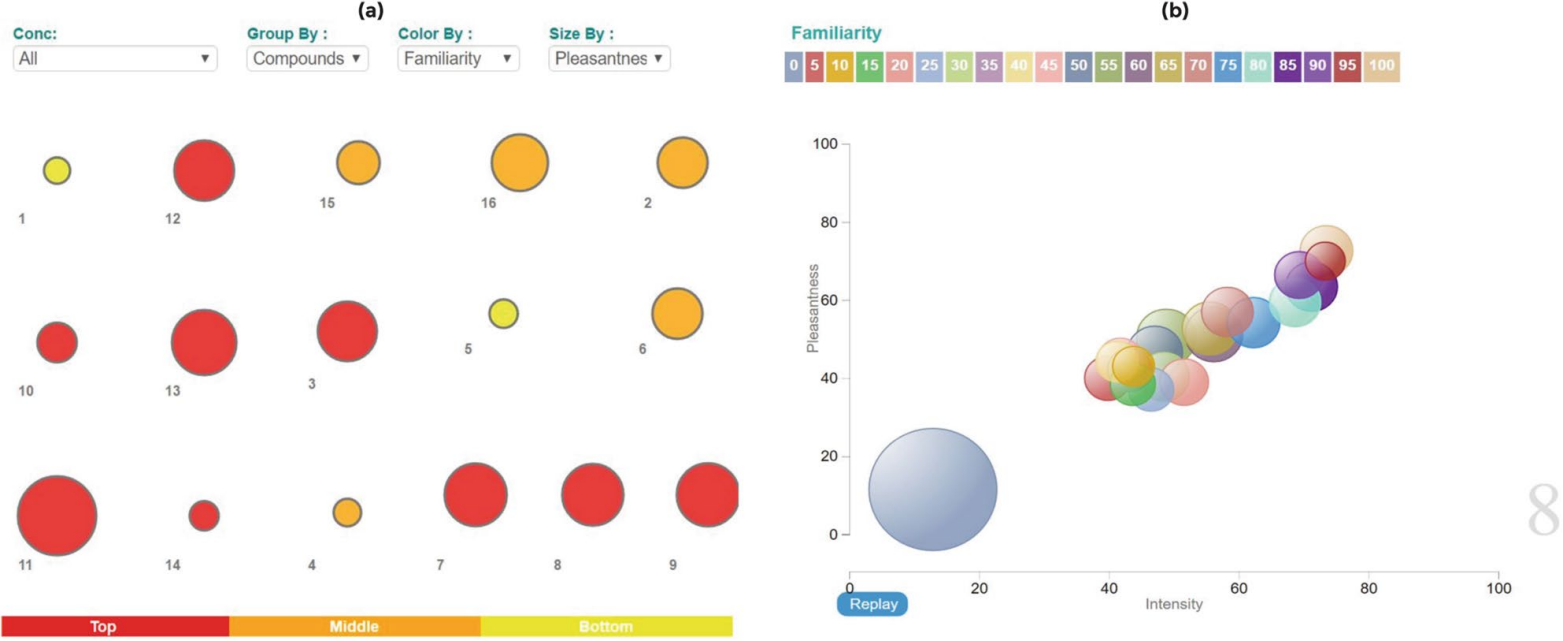
(**a**) Visualization of the data based on functional groups present (grouped by compounds), colored based on the perceived average familiarity among the subjects and sized according to the average pleasantness of the odor among the subjects. (**b**) Visualization of the ratings within each group (screenshot of group 8 representing aliphatic esters) showing the pattern of perceptual ratings in terms of familiarity, pleasantness and intensity. The bubble size represents the count of samples for each combination. (Group details are as follows, 1: aliphatics, 2: aromatics, 3: cyclic compounds, 4: alphatic acids, 5: aromatic acids, 6: aliphatic alcohols, 7: aromatic alcohols, 8: aliphatic esters, 9: aromatic esters, 10: aliphatic aldehydes, 11: aromatic aldehydes, 12: aliphatic ketones, 13: aromatic ketones, 14: organosulphur compounds, 15: nitrogen containing compounds, 16: others). Interactive charts for the above figures as obtained using TCS Vitellus are provided as additional information.

The raw data consists of 55,000 entries of perceptual data of 480 molecules at two dilutions. In order to analyze such a large dataset, TCS Vitellus (v2.2), an advanced data visualization platform was utilized to gain valuable insights from the complex data. The molecules were first grouped into 16 clusters based on the common functional group present such as ester, aldehyde, ketone, alcohol, carboxylic acid group, saturated cyclic structure and presence of nitrogen or sulphur atoms. The perceptual ratings ranging from 0 to 100 pertaining to odor intensity, pleasantness and familiarity at a dilution of 1/1000 were discretized into 21 batches (0–5, 5–10, 10–15 and so on). Figure 1 shows the trends observed across the clusters in their average values of perceived pleasantness and familiarity. It is observed that aromatic aldehydes were rated to be the most pleasant and the most familiar compounds. On the other hand, open-chained aliphatic compounds and aromatic carboxylic acids were the least pleasant and the least familiar odorant compounds. Compounds containing sulphur were observed to be the least pleasant among the familiar odors. This observation is in line with the fact that organosulphur compounds are often associated with a foul smell. Groups that were perceived to be less familiar but more pleasant can be explored for formulating new fragrances. Furthermore, the groups with least average familiarity and pleasantness could be possible starting points of research for molecule design in order to improve their hedonic attributes.

An in-depth analysis of the data for each of the groups depicted that the majority of the data points corresponded to low values of intensity, pleasantness and familiarity. It was observed that subjects have predominantly given lower pleasantness ratings for unfamiliar smells across the groups. For aromatic aldehydes and nitrogen containing compounds approximately 20% data points exhibited a reduction in the average pleasantness ratings with the increase in average intensity ratings. This effect was more pronounced in the case of aliphatic aldehydes, open-chained aliphatics and aromatic carboxylic acids where 69%, 49% and 44% data points respectively showed reduced pleasantness with increase in intensity. In general, across most groups, familiarity rating of 85 and above correspond to higher pleasantness ratings as well except for open-chained aliphatics, carboxylic acids, aliphatic aldehydes and aliphatic ketones. Such insights are useful in further interpretation of the predictive models.

### Data preprocessing for classification task

Classification being a supervised learning approach, mandates the availability of ground truth labels for the training data. The raw odor perception data however was not in a form that could be readily used to train classification algorithms. For the purpose of training and subsequent testing of a classification algorithm, each of the odorant compounds were assigned ground truth labels for their odor quality based on the frequency of usage of a semantic descriptor among the 55 subjects. Frequency of the descriptor usage for assigning representative odor quality of a compound is one of many such ways possible for assigning the ground truth. By using frequency as the deciding criterion, the subjects deviating from the population behavior are thereby excluded from consideration. The three most frequently used semantic descriptors among the 55 subjects were considered to be the odor quality labels for a given compound. Top three labels were chosen as opposed to a single label to take into account complexity of odor perception and the possibility that an odorant can be perceived to have multiple odor characters associated with it. Therefore, each odorant was represented by three odor characters (OC) associated with it. For example, ambroxan was represented by “sweet”, “flower” and “warm” odor characters at 1/1,000 dilution. Similarly, butylamine was perceived to have “grass”, “musky” and “spices” odor characters at 1/1,00,00,000 dilution. This gave rise to a complex multi-label classification problem which was solved by converting it to several binary classification models.

**Figure 2:**
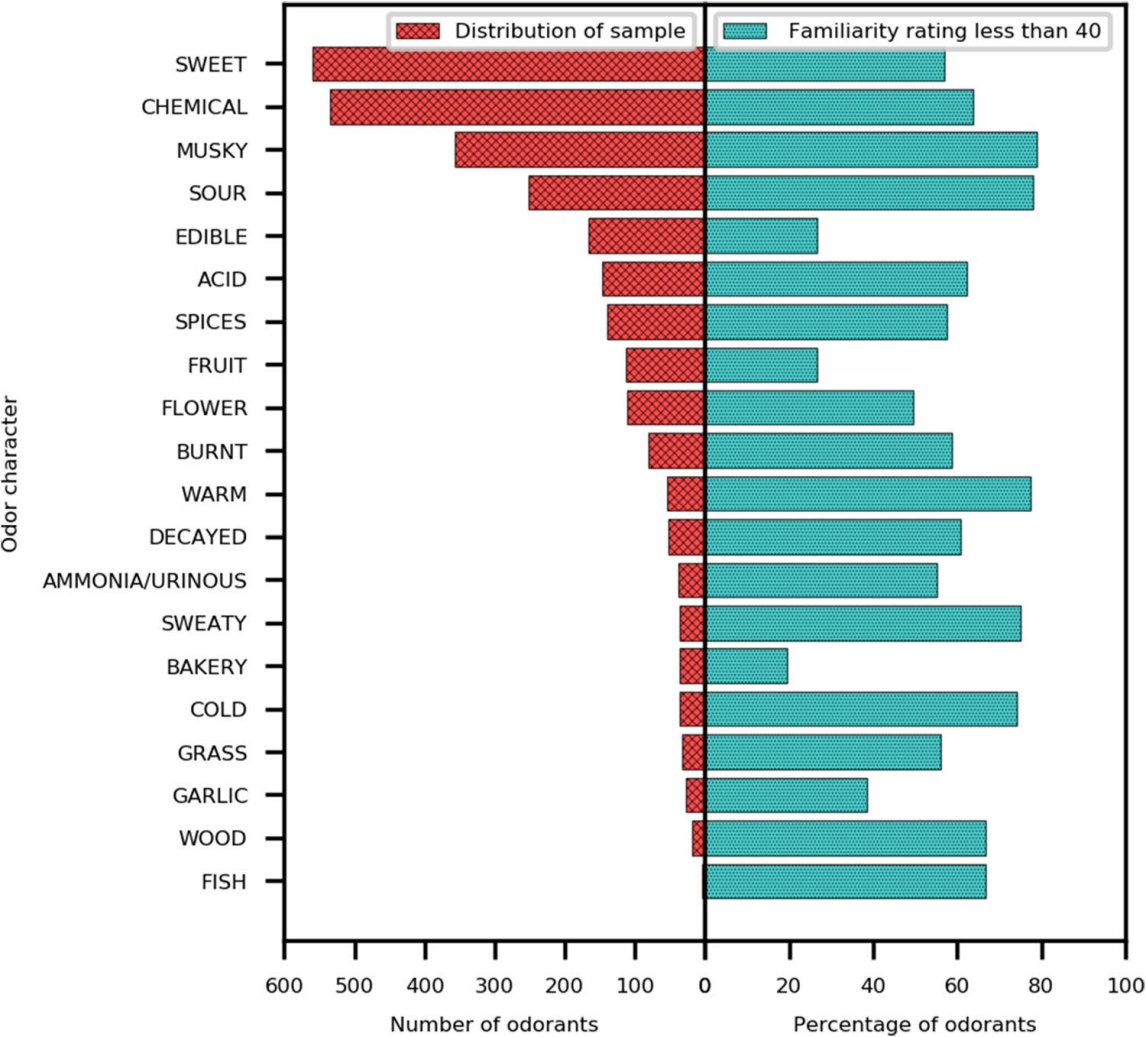
Distribution of samples associated with the odor character (left) and the percentage of odorants given low familiarity ratings by the subjects for each odor character (right).

Figure 2 shows the class distribution of the processed data after assigning the ground truth labels at the two dilutions. It demonstrates that maximum number of compounds (60.2%) were associated with having “sweet” OC, followed by “chemical” (57.6%) and “musky” (38.3%) OCs. As observed earlier, subjects were prone towards rating a lower value of pleasantness for unfamiliar smells. Figure 2 also shows the percentage of odorants rated to be less familiar by the subjects for each odor character. Approximately 78% of the compounds that were found to be associated with musky odors were given low familiarity ratings (less than 40). On the other hand, only 19% of the compounds associated with bakery odor character were given low familiarity ratings. Keller et al. also concluded that the descriptor “chemical” was used often when the subjects were unfamiliar with the smell^38^ which is supported by the observation that 63.7% compounds perceived to have “chemical” OC are rated to be unfamiliar by the majority of the subjects. The figure hints towards the existing pattern in the data that subjects when profiling unfamiliar smells were prone to choosing semantic descriptors such as “musky”, “sour”, “warm”, “sweaty” and “cold”.

### Model development

#### Structural features of molecules

In this study 196 two-dimensional RDKit molecular descriptors were used as input features for training machine learning algorithms for classification of odor characters. RDKit descriptors are divided into topological descriptors, connectivity descriptors, constitutional descriptors, molecular property and MOE-type descriptors. These descriptors provide quantitative information about the physical, chemical and topological characteristics of a molecule calculated from the 2D graph representations of the structures. Topological descriptors characterize the molecules based on their overall shape, size and degree of branching. Connectivity descriptors developed by Kier and Hall^40^ include chi indices which, encode the atomic and valence state electronic information and kappa indices characterize the molecular shape. Constitutional descriptors are based on simple counts of molecular features, such as atom counts, functional group counts, rotatable bonds, hydrogen bond acceptors and hydrogen bond donors. Molecular weight, log P and molar refractivity fall under the umbrella of molecular property type descriptors. MOE-type descriptors are the descriptors implemented in the Molecular Operating Environment (MOE) software, considering surface area contributions to molecular properties, such as partial charge and log P. The compound identification (CID) number of 480 odorants provided in the original dataset was used to generate the SMILES notation of the molecules which was subsequently utilized to calculate the RDKit descriptors. Some of the descriptor values were zero for all 480 molecules and were therefore removed, leaving 154 input structural features for model development.

#### Algorithms

**Figure 3:**
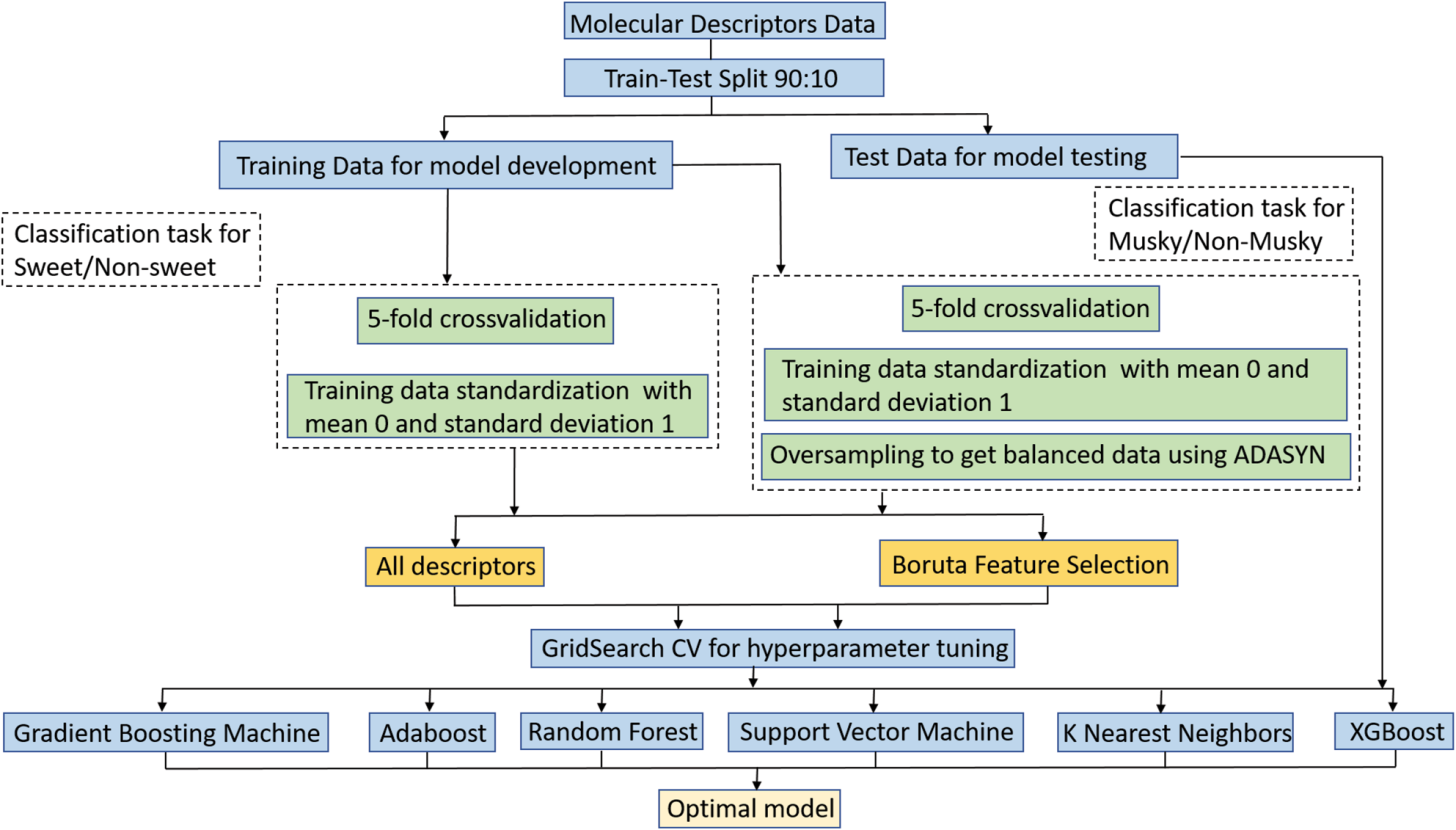
Overall workflow of model development for the classification tasks.

The overall workflow for the classification tasks is given in Fig. 3. One of the goals of using a data-driven model was to ascertain how does the perception of an odor character correlate with the structural features of a molecule. This approach requires that enough training data be available of the odorants associated with a given odor character under consideration. Based on the class distribution, it was observed that “sweet” and “chemical” OCs had enough samples for the positive class (60.17% and 57.6% respectively) while “musky” OC distribution (38.3% positive samples) was skewed towards the negative class. Other odor characters were not considered for model building due to high imbalance in their distribution. Predictive model for “chemical” OC was not attempted based on the observation made by Keller et al. that chemical semantic descriptor had only weak correlations with molecular features^38^. Therefore, binary classification models were built for “sweet” and “musky” OC only. The processed dataset was randomly split into a training and testing set with a 9:1 ratio. Only the training set was utilized for model parameter tuning by using a five-fold cross-validation approach to ensure generalizability. The testing set was used to report performance on unseen data and to choose the optimal model for the classification task. Several supervised learning algorithms were used to train models for both the classification tasks. The algorithms used included random forest classifier, gradient boosting, adaptive boosting (AdaBoost), extreme gradient boosting (XGBoost), support vector machine (SVM) and k-nearest neighbors (KNN) algorithm.

The dilution was considered to be a categorical input feature along with the 154 real-valued molecular features. After one-hot encoding of the categorical feature, we were left with a total of 158 input features for model training. The target variable was either 1 or 0 based on whether the compound belonged to the positive class or the negative class, respectively. For example, for the sweet OC classification task, vanillin was assigned 1 as target value, for it was associated with sweet OC while bis(methylthio)methane did not have sweet OC in its top three odor characters hence, was assigned a value of 0.

For both classification tasks, a five-fold cross-validation approach was followed to obtain a generalized model ensuring that the trained model does not overfit to the training data. During cross-validation, at every iteration the training set was split into five folds where one fold was kept as validation set to test the performance with the trained parameters. The four folds used for training were scaled to obtain a standard normal distribution. For the classification of “musky” OC, oversampling of the training data, excluding the validation set was done to reduce the imbalance in the data distribution. The comparison of the F1-scores of the “musky” OC classification model with and without oversampling is given in the Supplementary Table S1. It was observed that the models performed consistently better with oversampling. The model training involved the optimization of algorithm parameters such as, the number of decision trees, the depth of the trees and the minimum samples to split a node in case of random forest algorithm. F1-score, the harmonic mean of precision and recall was used as the performance metric for the combination of parameters during training. The best performing algorithm using 158 molecular features as inputs, was AdaBoost with a test F1-score of 0.825 for “sweet” OC and gradient boosting for “musky” OC with a test F1-score of 0.683. However, it has to be noted that some of the input features might be irrelevant for the classification tasks. Boruta, a feature selection algorithm was used to obtain relevant input features. Boruta algorithm is used to iteratively remove features that are statistically less relevant than random probes^41^. Boruta was preferred over other feature selection algorithms because it provides all-relevant features as opposed to only the minimal-optimal features provided by the other algorithms. Furthermore, it was undesirable to build a black box predictive model with minimal features to merely boost classification performance. Rather, a model that has features that are informative of the underlying mechanism of odor perception was required. The use of feature selection algorithms that use correlation between a feature and the target labels to find optimal features such as filter methods are undesirable in this context because lack of direct correlation between a feature and target is not proof that it is not important in conjunction with other variables.

**Figure 4:**
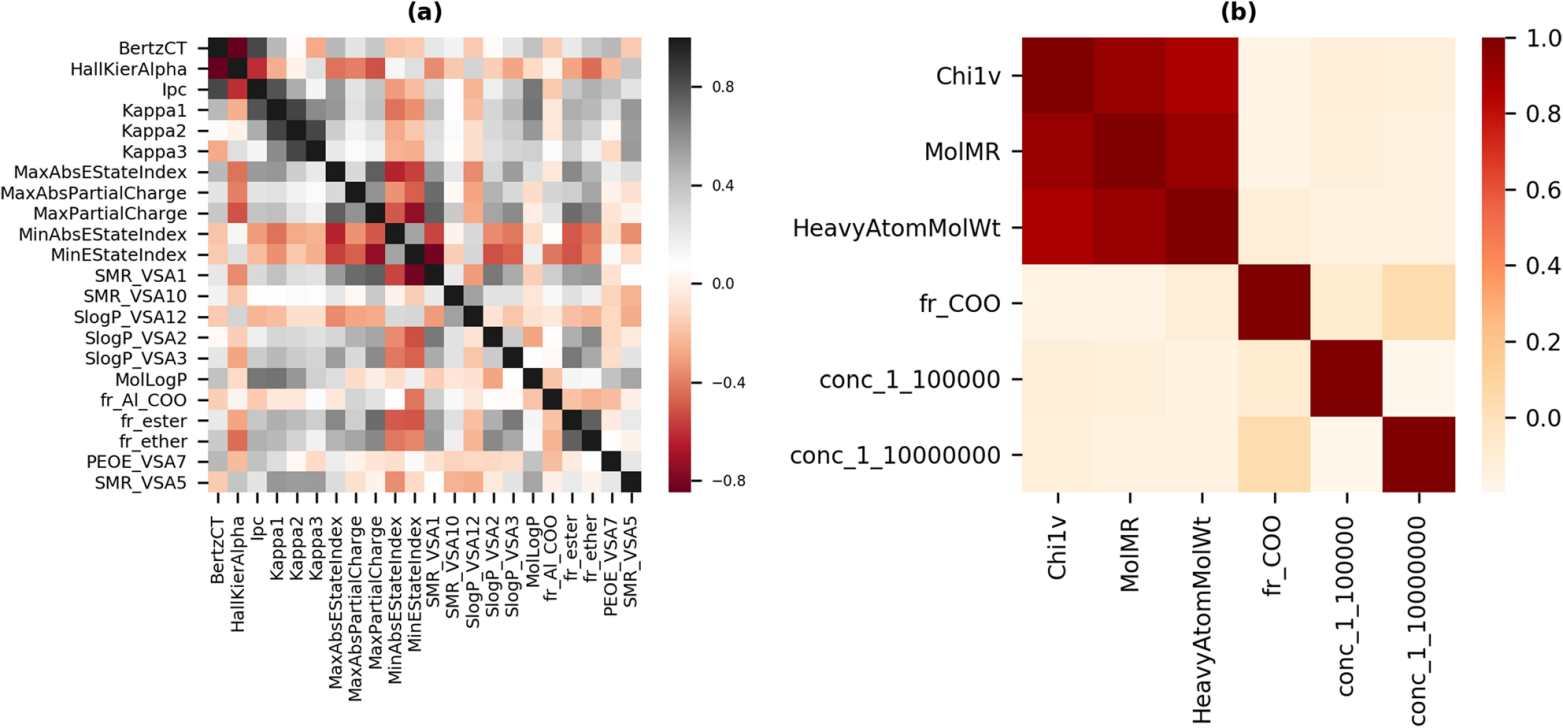
Heatmap showing correlation between features obtained after feature selection for (**a**) sweet OC prediction and (**b**) musky OC prediction.

After feature selection, we were left with 24 features for “sweet” OC and 6 features for “musky” OC. With the reduced features, random forest algorithm was the best performing model with a test F1-score of 0.824 in case of “sweet” OC classification (Table 1). There was a marginal increase in performance for “musky” OC with a test F1-score of 0.704 using gradient boosting algorithm and 0.697 using XGBoost algorithm (Table 2). Since Boruta uses correlation of feature with the target variable alone as the measure of relevance, there is possibility of presence of intercorrelation within the selected features. The heatmap of the reduced set of features showed that some of the features used are correlated to each other (Fig. 4). Therefore, feature selection was repeated after the removal of highly correlated features to compare performance. Highly correlated features were removed by calculating the spearman correlation coefficient between pairs of features. A threshold of 0.85 on the absolute value of the correlation coefficient was kept to remove one feature from the correlated pair. The removal of correlated features led to an increase in the performance of the sweet classification algorithms with XGBoost algorithm giving the maximum test F1-score of 0.84. The validation set performance was comparable to that of the training set showing that the model parameters are not overfitting to the training data. On the other hand, in case of “musky” OC a reduction in the F1-score was observed after feature selection with uncorrelated features. The reduction in performance could be attributed to lack of enough input information for discrimination of the classes as only three features were found to be statistically relevant after removal of highly correlated features. Tables 1 and 2 summarizes the performance of all the algorithms for both classification tasks.

**Table 1:** Performance of the algorithms on the sweet OC classification task: optimal model in bold.

Algorithm	Train F1-score	Validation F1-score	Test F1-score
**All non-zero rdkit features – 158**			
Gradient boosting machine	0.823	0.792	0.813
AdaBoost	0.8	0.784	0.825
Random forest	0.839	0.789	0.821
Support vector machine	0.764	0.739	0.792
XGBoost	0.804	0.783	0.819
K nearest neighbors	0.802	0.778	0.78
**Features obtained after feature selection using boruta – 24**			
Gradient boosting machine	0.81	0.78	0.812
AdaBoost	0.797	0.778	0.81
Random forest	0.806	0.786	0.824
Support vector machine	0.768	0.749	0.824
XGBoost	0.798	0.773	0.814
K nearest neighbors	0.789	0.758	0.803
**Uncorrelated features obtained after feature selection – 22**			
Gradient boosting machine	0.816	0.785	0.813
AdaBoost	0.789	0.768	0.823
Random forest	0.818	0.785	0.824
Support vector machine	0.754	0.732	0.781
**XGBoost**	**0.799**	**0.774**	**0.84**
K nearest neighbors	0.784	0.773	0.838

**Table 2:** Performance of the algorithms on the musky OC classification task; optimal model in bold.

Algorithm	Train F1-score	Validation F1-score	Test F1-score
**All non-zero rdkit features – 158**			
Gradient boosting machine	0.651	0.601	0.683
AdaBoost	0.624	0.598	0.634
Random forest	0.661	0.573	0.636
Support Vector Machine	0.623	0.591	0.659
XGBoost	0.540	0.520	0.620
K nearest neighbors	0.589	0.523	0.643
**Features obtained after feature selection using Boruta – 6**			
Gradient boosting machine	0.680	0.633	0.704
AdaBoost	0.646	0.619	0.689
Random forest	0.678	0.638	0.628
Support vector machine	0.650	0.628	0.681
**XGBoost**	**0.650**	**0.630**	**0.697**
K nearest neighbors	0.647	0.629	0.644
**Uncorrelated features obtained after feature selection – 3**			
Gradient boosting machine	0.590	0.581	0.571
AdaBoost	0.606	0.566	0.582
Random forest	0.608	0.562	0.564
Support vector machine	0.539	0.513	0.528
XGBoost	0.613	0.567	0.644
K nearest neighbors	0.559	0.560	0.580

### Discriminative features for prediction task

The optimal models and their training, validation and test F1-scores are given in Tables 1 and 2 for the prediction of the odor characters. XGBoost algorithm outperforms all the other algorithms based on higher test F1-score and comparable training and validation performance for both the tasks. The model parameters of the optimal models for the two classification tasks are as follows. For “sweet” OC the parameters were: learning rate = 0.11, maximum depth = 5, minimum child weight = 1, number of estimators = 15, gamma = 1.5, regularization parameter alpha = 15 and regularization parameter lambda = 1. For the “musky” OC the parameters were, learning rate = 0.7, maximum depth = 5, minimum child weight = 5, n estimators = 190, subsample = 0.9, regularization parameter alpha = 16 and regularization parameter lambda = 0.009. As mentioned earlier, it is important to obtain data-based models that are able to provide insights on the underlying mechanism of odor perception in addition to obtaining high classification performance.

**Figure 5:**
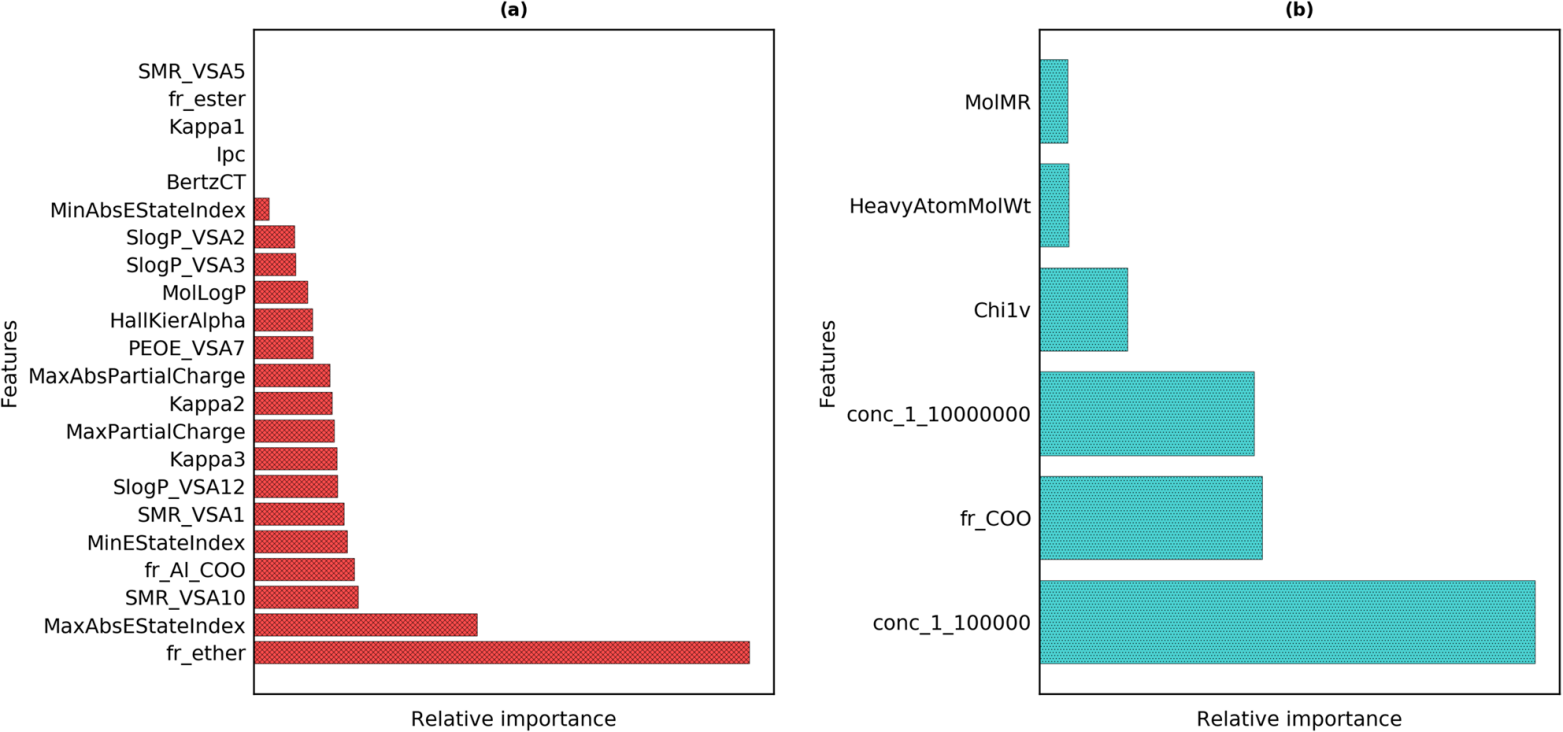
Input features ranked based on their importance in prediction of (**a**) sweet OC and (**b**) musk OC.

Figure 5 shows the feature importance values for the classification tasks as obtained for the optimal models suggestive of structural features that could assist in understanding the structure-odor character relationship. It is therefore essential to comprehend what physico-chemical attributes are conveyed by the descriptors. The chemical information conveyed by the features shown in Fig. 5 is given in the Supplementary Table S2. For the “sweet” OC, the most important feature for discriminating between the sweet and non-sweet class was the presence of ether functional groups in the molecule. MaxAbsEstateIndex, MinAbsEstateIndex and MinEstateIndex, types of electrotopological state (E-state) indices encode the electronic and the topological information of skeletal atoms in a molecule^42^. It is expressed as the intrinsic value of an atom modified due to the presence of other atoms in the molecule. Intrinsic value is derived from the count of p and lone-pair electrons, which is related to the valence-state electronegativity of the skeletal atom. The VSA descriptors are based on the atomic contributions to logP, molar refractivity and partial charge^43^. SlogP_VSA captures the hydrophobic and hydrophilic interactions, SMR_VSA used to represent the polarizability of a molecule and the direct electrostatic interactions are expressed through PEOE_VSA. These descriptors are fundamentally the sum of atomic van der Waals surface area (VSA) contributions of each atom to a property (SlogP, SMR and PEOE) in a specific range. For example, SMR_VSA1 is for the molar refractivity (MR) in the range (-∞,1.29). Kappa2 and kappa3 account for the spatial density of atoms and the centrality of branching in a molecule with the atom identity encoded through HallKierAlpha. Other relevant properties include the counts of aliphatic -COO group, maximum partial charge in the molecule and logP value calculated for the whole molecule. However, features such as BertzCT quantifying the complexity of the molecule, Ipc reflecting the branching in a molecule and kappa1 accounting for cyclicity were not among the relevant features. It is also widely known that the presence of ester groups is associated with “fruity” odors. It was also observed that the descriptors namely, count of ether groups, maximum absolute value of E-State index and SMR-VSA10 were among the top-five important features for all the tree-based algorithms (Supplementary Figures S1-S3). This indicates that along with the presence of ether functional group, the valence state electronegativity and the molar refractivity of the molecule affecting the van der Waals forces acting during molecule-receptor interaction are the most relevant for distinguishing between sweet and non-sweet classes. It is to be noted that a single feature alone or in pairs is not able to discriminate between the sweet and non-sweet class. However a distinction could be made with a combination of these features in the higher dimensional space. It is an interesting observation that dilution was not found to be statistically relevant for the “sweet“ OC classification task suggesting that the sweet perception of odorants used in this study is dilution-independent. Laing et al. investigated the effect of concentration on the odor qualities of oxygenated aliphatic compounds and found that four out five odorants had changes in odor quality with change in concentration^44^. However, it can also be observed that three of these odorants which had “sweet” odor quality, consistently possessed “sweet” odor quality among others even with changed concentration, while the other two odorants were not associated with “sweet” odor quality. It is to be noted that our models do not give information on how dominant is the odor character but that it is associated with the odorants by the subjects. Therefore, it is possible that with changes in dilution, the “sweet” odor character may not be the most dominating odor quality of the odorants, but can still possess some “sweet” character among others. This could possibly explain why “sweet” odor character is found to be dilution-independent in the present study. For “musky“ OC, the important features were dilution, counts of the carboxylic acid functional group, molecular weight, first-order chi connectivity descriptor and molar refractivity. The last two were also found to be the discriminative features among musks and non-musks in the study by Jurs and Ham^45^. The number of carboxylic acid groups as an important attribute has not been reported in the literature, however the presence of carbonyl group is deemed to be a necessary feature in macrocyclic musks^46^.

### Comparison of crowd perception with quality descriptors from other sources

The classification model for the prediction of “sweet” odor character performs fairly well on the unseen data. Nevertheless, we further analyzed the odorants misclassified by the model. The comparison of the odor description from Goodscents (https://www.thegoodscentscompany.com/) with the ground truth labels as obtained based on the subjects ratings for the misclassified compounds in the test set for sweet OC prediction task is given in Table 3. It demonstrates that within the misclassified set, the compounds with ground truth label of “sweet” were predominantly described with non-sweet semantic descriptors, with the exception of lepidine. For example, bis(methylthio)methane is described using “garlic”, “sulfurous”, “green”, “spicy” and “mushroom” odor characters.

**Table 3:** Odor description of the misclassified test compounds.

S. No	Molecule	Ground truth label	Predicted label	Familiarity rating	Organoleptics from goodscents
1	3-petanone	Non-sweet	Sweet	20	Ethereal acetone
2	allyl hexanoate	Non-sweet	Sweet	20	Sweet fruity pineapple tropical ethereal rum arrack fatty cognac
3	propyl acetate	Non-sweet	Sweet	40	Sweet and fruity
4	allyl phenyl acetate	Non-sweet	Sweet	40	Honey fruity rum
5	methyl – 3(methyl thio) propionate	Non-sweet	Sweet	20	Sulfurous vegetable onion sweet garlic tomato
6	1,6- hexalactam	Non-sweet	Sweet	20	Amine spicy
7	methyl (methyl thio) acetate	Non-Sweet	Sweet	60	Sulfurous cooked potato roasted nut fruity tropical
8	octyl isovalerate	Non-sweet	Sweet	20	Warm floral rose honey apple pineapple
9	Ambroxan	Non-sweet	Sweet	60	Ambergris old paper sweet labdanum dry
10	isobutyl alcohol	Non-sweet	Sweet	20	Ethereal winey
11	3-decen-2-one	Non-sweet	Sweet	20	Fatty green fruity apple earthy jasmine
12	benzaldehyde propylene glycol acetal	Non-sweet	Sweet	20	Bitter narcissus sweet napthalic woody
13	bis(methylthio)methane	Sweet	Non-sweet	20	Garlic sulfurous green spicy mushroom
14	trans-2-hexanal	Sweet	Non-sweet	20	Green leafy
15	2-(4-hydroxyphenyl)ethylamine	Sweet	Non-sweet	20	Meaty dirty cooked phenolic rubbery
16	2,5,-dimethyl pyrole	Sweet	Non-sweet	20	-
17	pyrazinyl ethane thiol	Sweet	Non-sweet	20	Sulfurous meaty cabbage
18	lepdidine	Sweet	Non-sweet	80	Burnt oil herbal floral sweet

**Figure 6:**
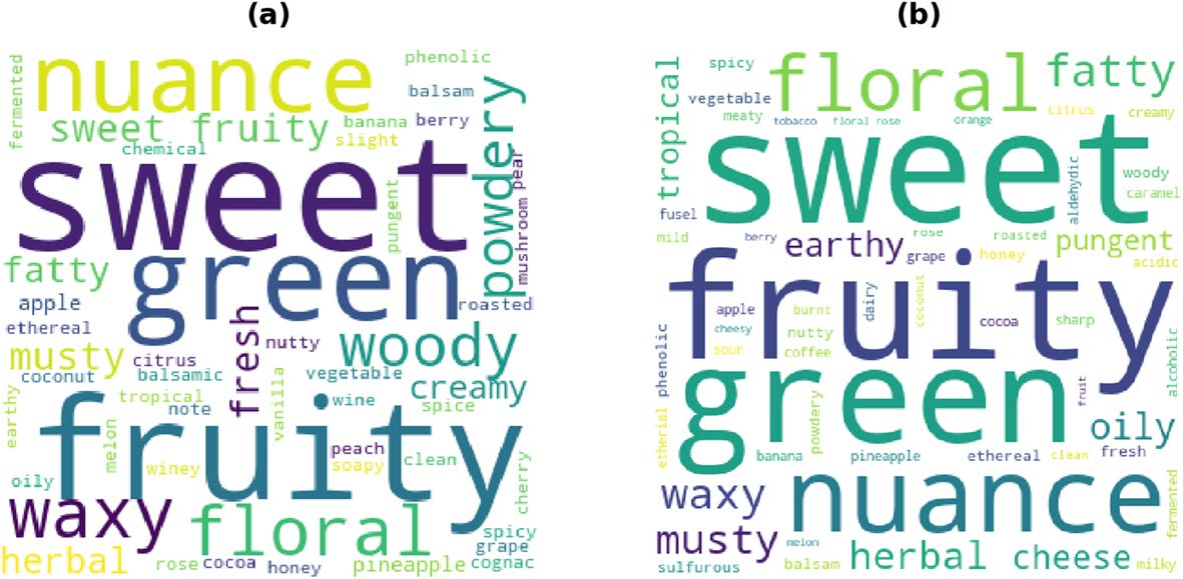
Word cloud of semantic descriptors reported for odorants labelled as (**a**) sweet and (**b**) musky.

Furthermore, most of the compounds labelled as non-sweet were described using either sweet or related semantic descriptors. For example, the odor description of allyl hexanoate consists of “sweet”, “fruity”, ’pineapple”, “tropical”, “ethereal”, “rum”, “arrack”, “fatty” and “cognac” while it was labelled to have “chemical”, “cold” and “musky” odor characters based on the subject ratings. This disparity in the odor quality description, coupled with the fact that these odorants were also given low familiarity ratings suggests that subjects were not able to relate the odor quality with the given semantic descriptors in the case of unfamiliar smells. One may argue if the compounds used for training showed similar discrepancy between the characters based on perceptual ratings and the odor description from other sources. The word cloud of the odor description for all the compounds with odor character label of “sweet” (Fig. 6a) shows that these compounds are in general associated with a sweet odor profile. Thus, it can be concluded that the misclassifications are as a result of the inherent bias towards unfamiliar odors in the data which the algorithm is not able to capture using just the molecular features. The analysis of the misclassified test set compounds for the “musky” OC did not show a similar pattern as observed with “sweet” OC (Supplementary Table S3). Additionally, the test F1-score for the “musky” OC prediction is lower as compared to the “sweet” model. This could be because the model is either unable to establish useful patterns in the data or that the molecular features do not correlate well with the ground truth. The word cloud of the corresponding published semantic descriptors for the odorants labelled to have “musky” odor character depicted that majority of the compounds are either described using words such as “sweet”, “green” and “fruity” (Fig. 6b). Furthermore, the word “musk” was used only twice to describe the odor of the compounds given a “musky” label. This indicates that the subjects were not able to associate the musky descriptor with a reference odor.

## Discussion

Machine learning algorithms applied to the domain of chemistry, are useful in determining chemical properties without resorting to extensive trial and error-based experimentation. The performance of these algorithms is extensively dependent on the data used for the training. There needs to be a good correlation between the input features and the ground truth labels and the data should have minimal outliers and noise. A data-driven model for odorant properties helps in prediction of odor character for novel molecules and can assist in understanding which structural parameters are relevant for an odorant exhibiting an odor quality. However, due to the subjective nature of odor perception any psychophysical study of odorants relating how a group of subjects perceive them needs to have appropriate measures to keep the study as objective as possible. We have attempted to bring objectivity in our study by considering the top-three odor qualities of the odorants associated by majority of the subjects in the psychophysical dataset of 55 subjects on 480 molecules. The visualization of the dataset revealed groups of compounds that could be explored to make new fragrance formulations. Simple data analytics such as these show that the presence or absence of a functional group in a molecule can be related to the hedonic attributes of the molecule. This is clearly seen in the case of organosulphur compounds where the presence of sulphur has a negative correlation with its pleasantness. It also provides possible candidates for redesigning molecules to improve their pleasantness. One clear insight from the visualization is that the perceptual ratings are biased towards the familiarity of the odors. This supports the theory that olfaction is a learned behaviour working in conjunction with the chemical properties of an odorant which would only determine which receptors will be activated by it. The subjects were prone towards rating lower values for intensity and pleasantness when the odors were unfamiliar. This was also observed in the usage of the semantic descriptors that the subjects had most associated with the unfamiliar smells. The semantic descriptors such as “musky”, “sour”, “warm”, “sweaty” and “cold” were predominantly associated with less familiar smells. The absence of a clear reference for the semantic descriptors could also be a factor for the subjects not being able to identify certain odors. This is evident in the difference in the performances of the “sweet” and “musky” predictive models. The comparison of the labels given by the subjects and experts reveal that most of the molecules that were perceived by the subjects to exhibit sweet smells were reported to be described using similar semantic descriptors. However, the molecules that the subjects in this study have associated with musky smells are described using descriptors that are not related to a musk fragrance. This could be due to the inability of the subjects to relate to “musky” as a semantic descriptor or due to their inexperience with traditional musky smells.

The model performance for the “sweet” OC show that features obtained from the two-dimensional structure of the molecules provide enough relevant information to obtain a good classification model using the decision tree-based ensemble techniques such as extreme gradient boosting also known as XGBoost. Decision tree-based methods work well when there is mix of continuous and categorical features. However, they can easily overfit to the training data and thus give a poor generalization performance. Therefore, crossvalidation is crucial to obtain a generalized model that can identify a general set of features that could help in distinguishing between classes, in this case the odor character. In order to establish the best performing model it is imperative that the test set contains unseen data which have not been utilized during any of the phases of model training, even when scaling the input features. Feature selection methods such as the Boruta algorithm, used in this study can help in overcoming the “curse of dimensionality” especially in such applications where the training data is usually limited. Imbalance in the data distribution of the classes can also lead to poor predictive performances hence oversampling methods have to be applied to negate the bias towards the majority class. It has to be noted that any oversampling performed should only include the training data and not on the validation or the test set.

The model trained on the “sweet” odor character data which had less noise and mislabeling compared to the data for the “musky” odor character, gave further insights on relevant features useful for the prediction task. These features include the basic structural features such as the count of ether functional groups and other derived features obtained from the chemical structure such as the EState indices and VSA descriptors which emphasize the atomic contributions towards properties like electronegativity, partial charge, and polarizability of the molecule. This is further supported by the observation that these descriptors were also among the important features for other tree-based models as well. Additionally, we found that “sweet” odor character is dilution-independent and it is possible that with changes in concentration, the “sweet” character is retained although it may not be the most dominant odor quality. We found that our trained model for the “sweet” odor character classification task is able to train well in spite of mislabeling in the data. The machine learning model predictions correlate with the labels associated by experts for these odorants which gives further evidence on the capability of the model to relate chemical information with the “sweet” odor character. The “musky” odor character classification model does not perform as well as the “sweet” model. This could be due to the extensive mislabeling in the data by the subjects and their bias towards unfamiliar odors. Therefore, structural parameters alone cannot capture the inherent bias in the data for the musky odors by the subjects. The unavailability of a reference odor for comparison during the study or lack of a memory or experience associated with musky smells is a plausible explanation for the bias. This observation suggests that for more objective results, the semantic descriptors chosen need to be given a proper reference so that valid data can be obtained as far as odor perception by untrained subjects are concerned. In conclusion, through this study we present that a useful machine learning based model relating the chemical features to the perceptual attributes can be obtained with data that has minimal bias from the subjects.

Psychophysical datasets give valuable insights on the differences in the perception of familiar and unfamiliar odors by untrained individuals. However, data-based methods for odor perception task suffer from bias and subjective interpretation of the semantic descriptors used during verbal profiling. Experts trained in the art of perfumery are able to recognize the nuances of odor quality of the odorant molecules thus giving a more objective data to train the models. In the “sweet” classification task the subjects had less misidentification and probably better understanding of what sweet odors are, thereby the algorithms performed fairly well on this data and even learnt the actual odor character of odorants that were probably mislabeled by the subjects. Therefore, having an objective data is important for training better models which could then point to better patterns in distinguishing the odor characters. Training a model on experts’ data could be one way of bringing objectivity in the data however crowd-sourcing the data is generally more feasible than performing such a large study with experts. In order to make better data-driven models using untrained subjects, a standardized protocol needs to be followed so that the data and the subsequent insights from the data are more reliable. For future work, inclusion of features relating to the subjects such as age, gender and cultural background can help in predicting how people sharing similar characteristics would perceive a smell in general.

## Methods

The SMILES notations of the odorant compounds were obtained using the “PubChemPy” package (version 1.0.4) of Python programming language. RDKit descriptors were calculated using ChemDes (version 3.0), a free web-based platform for calculation of molecular descriptors. All the steps for preprocessing of the data and model training were performed using Python libraries: NumPy^47^ (version 1.16.3), pandas^48^ (version 0.20.3), imblearn^49^ (version 0.0) and scikit-learn^50^ (version 0.21.2). GridSearchCV class was used to train and test the algorithms with crossvalidation. The search space for the parameters for the algorithms is provided in Supplementary Table S4. The “Pipeline” class of the imblearn package was used to scale and oversample the training data during the fivefold crossvalidation. For the tuning of the hyperparameters, an exhaustive grid-search for all possible combinations is computationally extensive with a large number of hyperparameters. Hence for tree-based methods such as Random forest, Gradient boosting and XGBoost, a coarse-search on parameters such as learning rate and number of estimators were first performed. Based on the highest average F1-score of the validation data, the values for the aforementioned parameter were kept fixed and other parameters such as max_depth, min_samples_split and max_features were varied to maximize the average F1-score. In case of XGBoost, parameters reg_alpha and reg_lambda were used for regularization of the weights to control overfitting. Other algorithms tuning such as adaboost, support vector machine and k-nearest neighbors were done using a grid-search approach, where a grid of parameter values were passed and models were trained and tested for all possible combinations of the parameters. Support Vector machine algorithm was trained using the “rbf” kernel. The parameters C and gamma were tuned using exponentially growing sequences of values. Feature selection was performed using the Boruta python package (version 0.1.5). The maximum number of iterations were set to be 500. For all the algorithms the random_state was set to a value of 1 for reproducibility. Figures are generated using Matplotlib^51^ (version 3.1.0), Seaborn (version 0.9.0) and wordcloud (version 1.5.0) libraries supported by Python programming language.

## Supplementary information

Supplementary file1

Supplementary file2

Supplementary file3

Supplementary file4

Supplementary file5

## References

[1] Reinarz J (2014). Past Scents: Historical Perspectives on Smell.

[2] Turin L, Sanchez T (2008). Perfumes: The AZ Guide.

[3] Toedt J, Koza D, Van Cleef-Toedt K (2005). Chemical Composition of Everyday Products.

[4] Sell CS (2006). The Chemistry of Fragrances: from Perfumer to Consumer.

[5] Desor J, Beauchamp GK (1974). The human capacity to transmit olfactory information. Percept. Psychophys..

[6] Ohloff G (1980). Stereochemistry-odor relationships in enantiomeric ambergris fragrances. Helv. Chim. Acta.

[7] Wise PM, Olsson MJ, Cain WS (2000). Quantification of odor quality. Chem. Senses.

[8] Engen T (1987). Remembering odors and their names. Am. Sci..

[9] Kaeppler K, Mueller F (2013). Odor classification: a review of factors influencing perception-based odor arrangements. Chem. Senses.

[10] Chastrette M (2002). Classification of odors and structure-odor relationships in *Olfaction, taste, cognition* 100–116.

[11] Yoshida M (1964). Studies of psychometric classification of odors (5). Jpn. Psychol. Res..

[12] Dravnieks A, Bock F, Powers J, Tibbetts M, Ford M (1978). Comparison of odors directly and through profiling. Chem. Senses.

[13] Cain WS (1982). Odor identification by males and females: predictions vs performance. Chem. Senses.

[14] Keller A, Hempstead M, Gomez IA, Gilbert AN, Vosshall LB (2012). An olfactory demography of a diverse metropolitan population. BMC Neurosci..

[15] Rabin MD (1988). Experience facilitates olfactory quality discrimination. Percept. Psychophys..

[16] Sell CS (2014). Chemistry and the Sense of Smell.

[17] Theimer ET (2012). Fragrance Chemistry: the Science of the Sense of Smell.

[18] Chastrette, M. & Zakarya, D. Molecular structure and smell in *The Human Sense of Smell,* 77–92 (Springer, Berlin, 1991).

[19] Amoore JE (1971). Stereochemical and vibrational theories of odour. Nature.

[20] Beets, M. G. J. *Structure-Activity Relationships in Human Chemoreception* (Applied Science Publishers, 1978).

[21] Amoore, J. E. *Molecular basis of odor* (Charles C Thomas, 1970).

[22] Chastrette M (1981). An approach to a classification of odours using physicochemical parameters. Chem. Senses.

[23] Polak E, Fetison G, Fombon AM, Skalli A (1988). Structure-odor relationships for “catty”-smelling mercapto compounds in humans. J. Agric. Food Chem..

[24] Witteveen, J. G. & van der Weerdt, A. J. Structure-odour relationships of some new synthetic sandalwood aroma chemicals: Synthesis and olfactive properties in a series of bicyclo[4.4.0]decan-3-ols. *Recl. Trav. Chim. Pays Bas* **106,** 29–34 (1987).

[25] Bushdid C, Magnasco MO, Vosshall LB, Keller A (2014). Humans can discriminate more than 1 trillion olfactory stimuli. Science.

[26] Mamlouk AM, Martinetz T (2004). On the dimensions of the olfactory perception space. Neurocomputing.

[27] Sell C (2006). On the unpredictability of odor. Angew. Chem. Int. Ed..

[28] Bentley R (2006). The nose as a stereochemist Enantiomers and odor. Chem. Rev..

[29] Egawa T, Kameyama A, Takeuchi H (2006). Structural determination of vanillin, isovanillin and ethylvanillin by means of gas electron diffraction and theoretical calculations. J. Mol. Struct..

[30] Leffingwell, J. C. et al. Olfaction–update no. 5. *Leffingwell reports* **2,** 1–34 (2002).

[31] Wilson DA, Stevenson RJ (2006). Learning to Smell: Olfactory Perception from Neurobiology to Behavior.

[32] Keller A (2017). Predicting human olfactory perception from chemical features of odor molecules. Science.

[33] Shang L, Liu C, Tomiura Y, Hayashi K (2017). Machine-learning-based olfactometer: prediction of odor perception from physicochemical features of odorant molecules. Anal. Chem..

[34] Zhang L, Mao H, Liu L, Du J, Gani R (2018). A machine learning based computer-aided molecular design/screening methodology for fragrance molecules. Comput. Chem. Eng..

[35] Luan F, Liu H, Wen Y, Zhang X (2008). Classification of the fragrance properties of chemical compounds based on support vector machine and linear discriminant analysis. Flavour Frag. J..

[36] Dravnieks A (1983). Odor character profiling. J. Air Pollut. Control Assoc..

[37] Licon, C. C. et al. Chemical features mining provides new descriptive structure-odor relationships. *PLoS Comput. Biol.* **15,** e1006945; 10.1371/journal.pcbi.1006945 (2019). 10.1371/journal.pcbi.1006945 PMC6504111 31022180

[38] Keller A, Vosshall LB (2016). Olfactory perception of chemically diverse molecules. BMC Neurosci..

[39] Li, H., Panwar, B., Omenn, G. S. & Guan, Y. Accurate prediction of personalized olfactory perception from large-scale chemoinformatic features. *GigaScience* **7,** gix127; 10.1093/gigascience/gix127 (2017). 10.1093/gigascience/gix127 PMC5824779 29267859

[40] Hall, L. H. & Kier, L. B. The molecular connectivity chi indexes and kappa shape indexes in structure-property modeling in *Reviews in computational chemistry, volume 2* 367–422 (Wiley-VCH, 1991).

[41] Kursa MB, Rudnicki WR (2010). Feature selection with the boruta package. J. Stat. Softw..

[42] Hall LH, Mohney B, Kier LB (1991). The electrotopological state: structure information at the atomic level for molecular graphs. J. Chem. Inf. Comput. Sci..

[43] Labute P (2000). A widely applicable set of descriptors. J. Mol. Graph. Model..

[44] Laing DG, Legha PK, Jinks AL, Hutchinson I (2003). Relationship between molecular structure, concentration and odor qualities of oxygenated aliphatic molecules. Chem. Senses.

[45] Ham CL, Jurs PC (1985). Structure-activity studies of musk odorants using pattern recognition: monocyclic nitrobenzenes. Chem. Senses.

[46] Rossiter KJ (1996). Structure-odor relationships. Chem. Rev..

[47] Van Der Walt S, Colbert SC, Varoquaux G (2011). The NumPy array: a structure for efficient numerical computation. Comput. Sci. & Eng..

[48] McKinney, W. Data structures for statistical computing in python. *Proc. 9th Python Sci. Conf.* **445,** 51–56 (2010).

[49] Lemaître G, Nogueira F, Aridas CK (2017). Imbalanced-learn: A python toolbox to tackle the curse of imbalanced datasets in machine learning. J. Mach. Learn. Res..

[50] Pedregosa F (2011). Scikit-learn: Machine learning in Python. J. Mach. Learn. Res..

[51] Hunter JD (2007). Matplotlib: A 2D Graphics Environment. Computing in Science and Engineering.

