# Supplementary material for: Data based predictive models for odor perception
Source: Sci Rep. 2020 Oct 13;10:17136. doi: 10.1038/s41598-020-73978-1 (PMC7553929; doi:10.1038/s41598-020-73978-1)
Supplement: Supplementary file 1 — Supplementary file1 [file 41598_2020_73978_MOESM1_ESM.docx]

**Data Based Predictive Models for Odor Perception**

**Rinu Chacko, Deepak Jain*, Manasi Patwardhan, Abhishek Puri, Shirish Karande and Beena Rai**

Tata Research Development and Design Centre, Tata Consultancy Services, 54-B, Hadapsar Industrial Estate,

Pune-411028, India

*Corresponding author: deepak.jain3@tcs.com

**Table S1:** Model performance for “Musky” odor character with and without using oversampling

| Algorithm | **Without Oversampling** | | | **With Oversampling** | | |
| --- | --- | --- | --- | --- | --- | --- |
|  | **Train F1 score** | **Validation F1 score** | **Test F1 score** | **Train F1 score** | **Validation F1 score** | **Test F1 score** |
| Gradient Boosting Machine | 0.56 | 0.5 | 0.59 | 0.68 | 0.633 | 0.704 |
| Adaboost | 0.45 | 0.4 | 0.48 | 0.646 | 0.619 | 0.689 |
| Random Forest | 0.61 | 0.496 | 0.613 | 0.678 | 0.638 | 0.628 |
| Support Vector Machine | 0.65 | 0.63 | 0.666 | 0.65 | 0.628 | 0.681 |
| XGBoost | 0.5 | 0.44 | 0.64 | 0.65 | 0.63 | 0.697 |
| K Nearest Neighbours | 0.529 | 0.501 | 0.6 | 0.647 | 0.629 | 0.644 |

**Table S2:** Chemical information conveyed by the descriptors used in the optimal models

| S. No. | Descriptor | Chemical information |
| --- | --- | --- |
| 1 | fr_ether | Count of ether functional groups |
| 2 | fr_Al_COO | Count of aliphatic carboxylic acids |
| 3 | fr_ester | Count of ester functional groups |
| 4 | fr_COO | Count of carboxylic acid groups |
| 5 | MaxAbsEStateIndex | Maximum absolute E-state value;  The E-state value for a given non-hydrogen atom in a molecule is given by its intrinsic state plus the sum of the perturbations on that atom from all the other atoms in a molecule. |
| 6 | MinEStateIndex | Minimum E-State value |
| 7 | MinAbsEstateIndex | Minimum Absolut E-State value |
| 8 | MaxPartialCharge | Maximum value of partial charge |
| 9 | MaxAbsPartialCharge | Maximum absolute value of partial charge |
| 10 | SMR_VSA10 | Intended to capture polarizability;  It is characterized as the amount of surface area with molar refractivity in the range [4.0, ∞) |
| 11 | SMR_VSA5 | It is characterized as the amount of surface area with molar refractivity in the range [2.45, 2.75) |
| 12 | SMR_VSA1 | It is characterized as the amount of surface area with molar refractivity in the range (-∞, 1.29) |
| 13 | SlogP_VSA12 | Intended to capture hydrophobic and hydrophilic effects;  It is characterized as the amount of surface area with logP in the range [0.6, ∞) |
| 14 | SlogP_VSA3 | It is characterized as the amount of surface area with logP in the range [-0.20, 0.0) |
| 15 | SlogP_VSA2 | It is characterized as the amount of surface area with logP in the range [-0.40, -0.20) |
| 16 | PEOE_VSA7 | Intended to capture direct electrostatic interation;  It is characterized as the amount of surface area with partial charge in the range [-0.05, 0.0) |
| 17 | kappa3 | It encodes information about the centrality of branching in a molecule. |
| 18 | kappa2 | It encodes information about the spatial density of atoms in a molecule. |
| 19 | kappa1 | It encodes the cyclicity of a molecule. |
| 20 | HallKierAlpha | It is used to encode atom identity in the kappa indices |
| 21 | MolLogP | Wildman-Crippen LogP value; captures the hydrophobicity of the molecule |
| 22 | MolMR | Wildman-Crippen Molar Refractivity value; captures the polarizability of the molecules |
| 23 | BertzCT | It quantifies the complexity of molecules by taking into account the size, symmetry, branching, rings, multiple bonds and heteroatom characteristic of a complex molecule. |
| 24 | Ipc | It reflects the branching in a molecule |
| 25 | Chi1v | It quantifies the molecular structure based on the topological and electronic character of the atoms in the molecule |
| 26 | HeavyAtomMolWt | The average molecular weight ignoring the hydrogen atoms |

**Table S3:** The odor description as obtained from Goodscents for the misclassified test set compounds corresponding to the musky odor character.

| **S.No.** | **Molecule** | **Ground Truth Label** | **Predicted Label** | **Familiarity Rating** | **Organoleptics from Goodscents** |
| --- | --- | --- | --- | --- | --- |
| 1 | Iodoform | Musky | Non-Musky | 60 | NA |
| 2 | pentan-1-ol | Musky | Non-Musky | 60 | Pungent, fermented, bready, yeasty, fusel, winey and solvent-like |
| 3 | neryl acetate | Musky | Non-Musky | 60 | floral rose soapy citrus dewy pear |
| 4 | 6-methylcoumarin | Musky | Non-Musky | 80 | Sweet coconut, vanilla, creamy with a powdery floral nuance |
| 5 | Phenoxyethyl isobutyrate | Musky | Non-Musky | 80 | sweet fruity tropical rose honey floral waxy |
| 6 | 1-phenyl-1-propanol | Non-Musky | Musky | 20 | sweet floral balsam |
| 7 | Pyridine | Non-Musky | Musky | 20 | sour fishy ammoniacal |
| 8 | Acetaldehyde | Non-Musky | Musky | 20 | pungent ethereal aldehydic fruity |
| 9 | 5-methyl quinoxaline | Non-Musky | Musky | 60 | toasted roasted corn nutty coffee burnt |
| 10 | dimethyl trisulfide | Non-Musky | Musky | 100 | sulfurous cooked onion savory meaty |
| 11 | decanal dimethyl acetal | Non-Musky | Musky | 20 | waxy herbal citrus green neroli |
| 12 | methyl phenyl acetate | Non-Musky | Musky | 80 | sweet floral honey spice waxy almond |
| 13 | phenethyl alcohol | Non-Musky | Musky | 100 | floral rose dried rose flower rose water |
| 14 | trans-5-methyl-2-hepten-4-one | Non-Musky | Musky | 20 | hazelnut nutty |
| 15 | piperonyl isobutyrate | Non-Musky | Musky | 20 | fruity berry jam plum tropical heliotrope powdery |
| 16 | L-glutamine | Non-Musky | Musky | 20 | milky custard cocoa oily |
| 17 | para-dimethyl hydroquinone | Non-Musky | Musky | 20 | sweet green new mown hay fennel |
| 18 | 4-methyl-5-vinylthiazole | Non-Musky | Musky | 20 | musty nutty root vegetable cocoa |
| 19 | 2-isopropylphenol | Non-Musky | Musky | 20 | solvent like, phenolic, smoky with toasted woody and burnt rubber nuances |
| 20 | 2,3-butanedione | Non-Musky | Musky | 20 | strong butter sweet creamy pungent caramel |
| 21 | allyl 2-ethylbutyrate | Non-Musky | Musky | 20 | nut fruit peach-pit cherry-pit ethereal |
| 22 | 2-methyl furan | Non-Musky | Musky | 20 | ethereal acetone chocolate |
| 23 | cyclopentyl mercaptan | Non-Musky | Musky | 100 | alliaceous onion garlic horseradish vegetable celery eggy |
| 24 | hexyl octanoate | Non-Musky | Musky | 20 | fruity green waxy berry apple ester |
| 25 | 2-acetylthiophene | Non-Musky | Musky | 60 | sulfurous nutty hazelnut walnut |
| 26 | 4-heptanone | Non-Musky | Musky | 20 | fruity cheese sweet cognac pineapple |
| 27 | 2,4,5-trimethylthiazole | Non-Musky | Musky | 20 | musty nutty vegetable cocoa hazelnut chocolate coffee |

**Table S4:** Grid search parameters used for model tuning

| **S. No.** | **Algorithm** | **Parameters** | **Search Space** |
| --- | --- | --- | --- |
| 1 | K Nearest Neighbors (KNN) | weights | {‘uniform’, ‘distance’} |
|  |  | p | {1, 2, 3} |
|  |  | n_neighbors | [2, 30] |
| 2 | Support Vector Machine | kernel | {‘linear’, ‘rbf’} |
|  |  | gamma | [2e-7, 2e3] |
|  |  | C | [2e-3, 2e7] |
| 3 | XGBoost | max_depth | [2, 5] |
|  |  | min_child_weight | [1, 6] |
|  |  | gamma | [0.1, 10] |
|  |  | subsample | [0.6, 1] |
|  |  | colsample_bytree | [0.5, 1] |
|  |  | reg_alpha | [0.1, 20] |
|  |  | reg_lambda | [0.001, 100] |
|  |  | learning_rate | [0.01, 1.0] |
|  |  | n_estimators | [10, 200] |
| 4 | Random Forest | criterion | {‘gini’, ‘entropy’} |
|  |  | n_estimators | [50, 100] |
|  |  | max_depth | [2, 5] |
|  |  | min_samples_split | [2, 70] |
|  |  | min_samples_leaf | [1, 50] |
| 5 | Adaboost | learning_rate | [0.1, 1.0] |
|  |  | n_estimators | [10, 100] |
|  |  | algorithm | {‘SAMME’, ‘SAMME.R’} |
| 6 | Gradient Boosting Machine (GBM) | learning_rate | [0.05, 1.0] |
|  |  | n_estimators | [10, 100] |
|  |  | max_depth | [2, 5] |
|  |  | min_samples_split | [10, 200] |
|  |  | min_samples_leaf | [30, 70] |
|  |  | max_features | [8, 159] |
|  |  | subsample | [0.6, 1.0] |


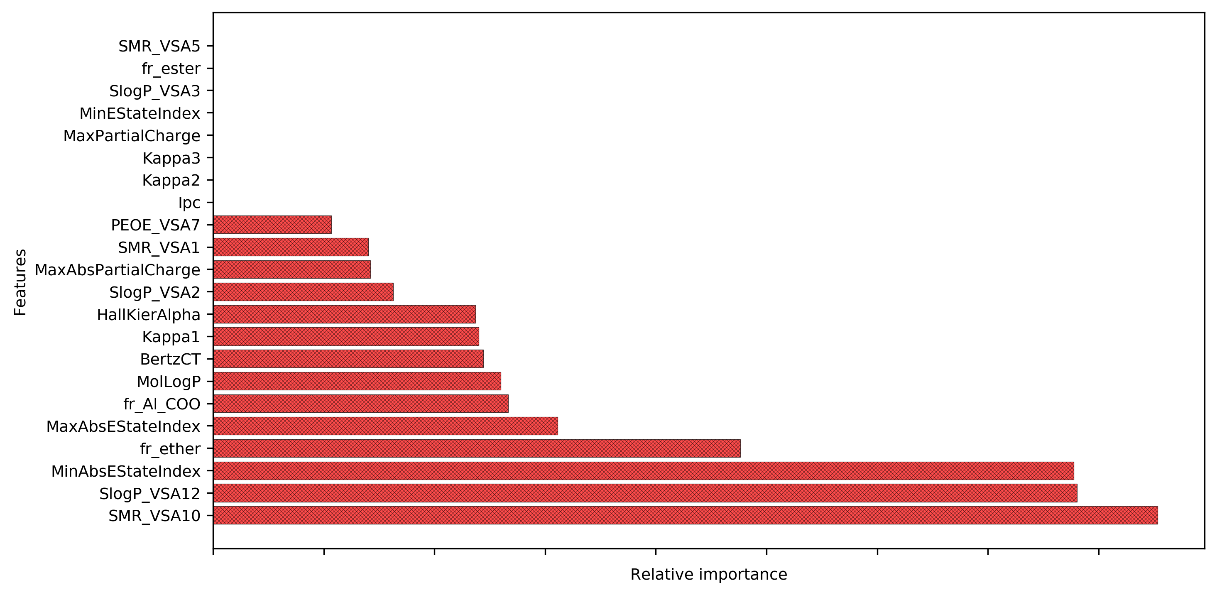


**Figure S1**. Input features ranked based on their importance in prediction of Sweet OC using Adaboost algorithm


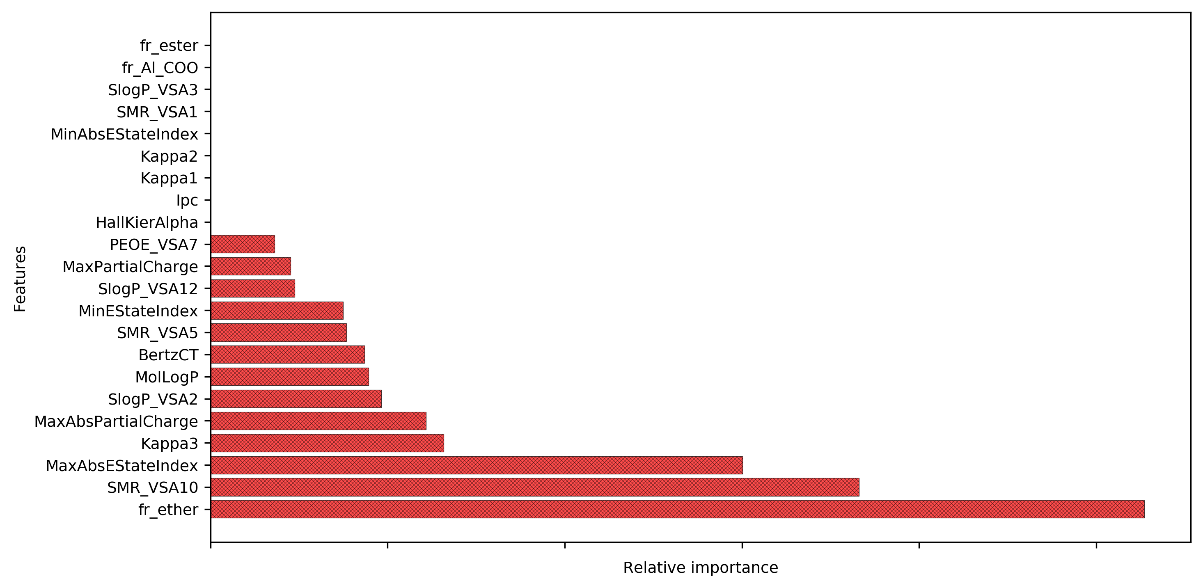


**Figure S2**. Input features ranked based on their importance in prediction of Sweet OC using GBM algorithm


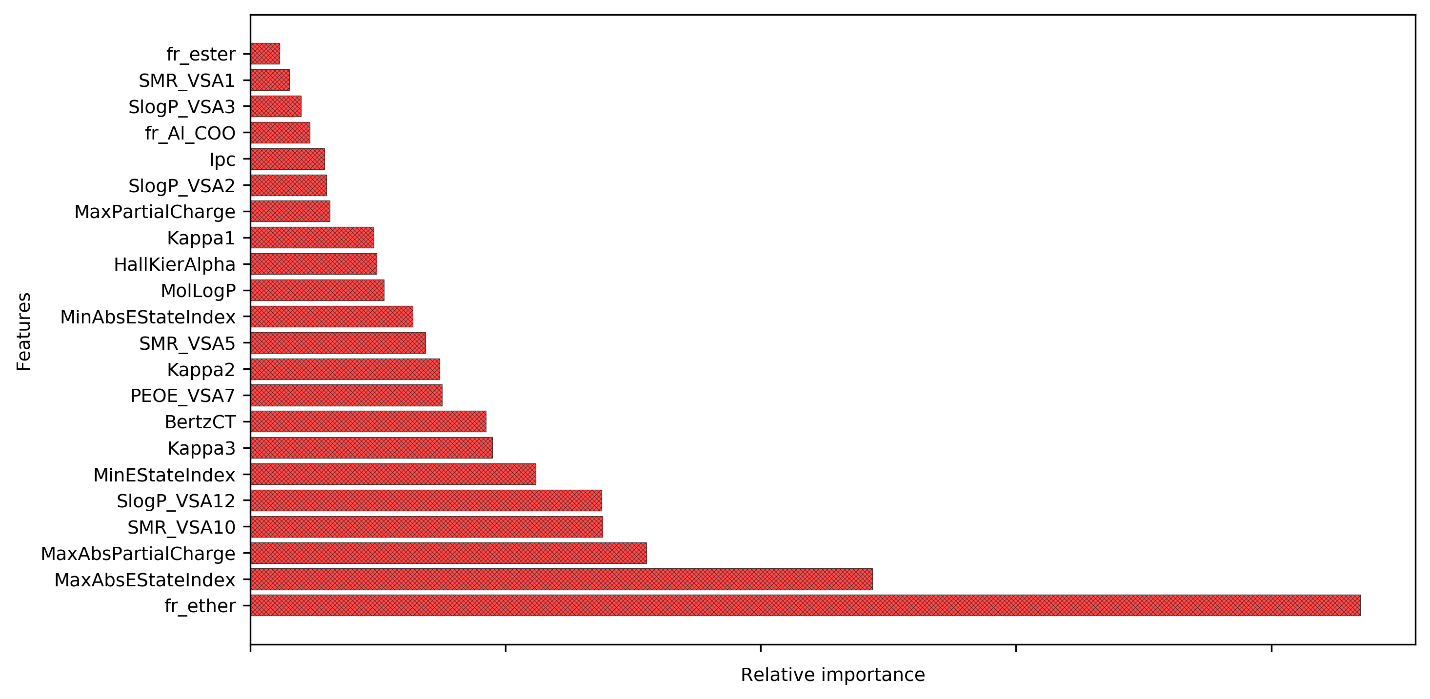


**Figure S3**. Input features ranked based on their importance in prediction of Sweet OC using random forest algorithm
