# Supplementary material for: Data based predictive models for odor perception
Source: Sci Rep. 2020 Oct 13;10:17136. doi: 10.1038/s41598-020-73978-1 (PMC7553929; doi:10.1038/s41598-020-73978-1)
Supplement: Supplementary file 3 — Supplementary file3 [file 41598_2020_73978_MOESM3_ESM.zip › .svn/text-base/chart.html.svn-base]

Copyright � 2016, Tata Consultancy Services Limited. All Rights Reserved. Terms of Use.
Document ID: CGSL000404
