# Supplementary material for: Data based predictive models for odor perception
Source: Sci Rep. 2020 Oct 13;10:17136. doi: 10.1038/s41598-020-73978-1 (PMC7553929; doi:10.1038/s41598-020-73978-1)
Supplement: Supplementary file 3 — Supplementary file3 [file 41598_2020_73978_MOESM3_ESM.zip › .svn/text-base/Copyright - NOTICES for Vitellus V 1.2.1.docx.svn-base]

# NOTICES AND INFORMATION

**TCS LICENSED PROGRAM: Vitellus**

**VERSION: 1.2.1**

(NOTE: These notices cover copyright notices and terms and conditions applicable to the non-TCS code, all or portions of which may be included Vitellus in TCS Licensed Program)

This file contains details concerning notices applicable to certain non-TCS code included in the TCS software product(s) listed above (each, a “Licensed Program"). In certain cases, Licensee may be referred to this file in the TCS License Agreement or by a specific reference elsewhere (for example, on a relevant web download page for TCS software products, or in the accompanying documentation).

Notwithstanding the terms and conditions of any other agreement that Licensee may have with Tata Consultancy Services Limited or any of its direct or indirect subsidiary companies (collectively "TCS"), use by Licensee (as part of Licensee’s use of the relevant TCS Licensed Program) of the non-TCS (third party) code components identified below is subject to the terms and conditions of the license agreement that applies to the respective non-TCS (third party) code contained in or referred to by any of the various notices below.

IMPORTANT NOTES AND DISCLAIMERS:

a) The notices set out below in this file are provided to Licensee for informational purposes.

b) This Notices file may include or identify information that in fact is not used by, or that was not shipped with, the Licensed Program as installed by Licensee.

c) TCS does not represent or warrant that the information in this NOTICES file is accurate.

d) Third party websites are independent of TCS, and TCS does not represent or warrant that the information on any third party web site referenced in this NOTICES file is accurate.

e) TCS disclaims any and all liability for errors and omissions, and for any damages accruing from the use of this NOTICES file or its contents, including (without limitation): (i) web site URLs; and (ii) any reference to a third party website.

SOURCE CODE AVAILABILITY

Licensee may be entitled to obtain the source code of specific individual components listed below; in any such case, Licensee may obtain the relevant source code from TCS by addressing an e-mail request to: [ujwala.nshahane@tcs.com](mailto:XXX.XXXX@tcs.com)

CONTENTS:-

THIS FILE CONTAINS NOTICES AND INFORMATION FOR VARIOUS SPECIFIC COMPONENTS, CATEGORISED UNDER THE FOLLOWING HEADINGS:-

**Licenses**

1. Apache License 2.0
2. MIT License
3. ISC License
4. BSD 3-clause "New" or "Revised" License
5. Microsoft Public License
6. GNU Lesser General Public License v2.1 or later
7. Common Development and Distribution License 1.0

- **Name of the components Detailed license terms with copyright notices**

**The Licensed Program includes all or portions of the following software which TCS obtained under the terms and conditions of the open source license mentioned in the table below:**

|  | | |  |  |  |  |  |
| --- | --- | --- | --- | --- | --- | --- | --- |
| **Component** | **Version** | **Home Page** | **License** | **License URL** | **Usage** | **Ship Status** | **Attribution Note** |
| Apache Commons Collections | 3.2.1 | <http://commons.apache.org/collections/> | Apache License 2.0 | http://www.apache.org/licenses/LICENSE-2.0 | File | Ship | Copyright © 2016 The Apache Software Foundation |
| Apache Log4j | 1.2.12 | <http://logging.apache.org/log4j/1.2/> | Apache License 2.0 | http://www.apache.org/licenses/LICENSE-2.0 | File | Ship | Copyright © 2016 The Apache Software Foundation |
| Apache Nutch |  | <http://lucene.apache.org/nutch/> | Apache License 2.0 | http://www.apache.org/licenses/LICENSE-2.0 | File | Ship | Copyright © 2016 The Apache Software Foundation |
| Apache Rave |  | <http://rave.apache.org/> | Apache License 2.0 | http://www.apache.org/licenses/LICENSE-2.0 | File | Ship | [Copyright © 2012 The Apache Software Foundation, Licensed under the Apache License, Version 2.0.](http://www.apache.org/licenses/LICENSE-2.0) |
| Apache Spark |  | <https://spark.apache.org/> | Apache License 2.0 | http://www.apache.org/licenses/LICENSE-2.0 | File | Ship | [Copyright © 2016 The Apache Software Foundation, Licensed under the Apache License, Version 2.0.](http://www.apache.org/licenses/LICENSE-2.0) |
| Apache XMLBeans | 2.3.0 | <http://xmlbeans.apache.org/> | Apache License 2.0 | http://www.apache.org/licenses/LICENSE-2.0 | File | Ship | Copyright © 2004 The Apache Software Foundation. |
| Apache-Jakarta BeanUtils | 1.8.0 | <http://jakarta.apache.org/commons/beanutils/index.html> | Apache License 2.0 | http://www.apache.org/licenses/LICENSE-2.0 | File | Ship | [Copyright © 2000-2014 The Apache Software Foundation. All Rights Reserved.](http://www.apache.org/) |
| Apache-Jakarta Codec | 1.4 | <http://jakarta.apache.org/commons/codec/> | Apache License 2.0 | http://www.apache.org/licenses/LICENSE-2.0 | File | Ship | [Copyright © 2016 The Apache Software Foundation](http://www.apache.org/licenses/LICENSE-2.0) |
| bizmoso | trunk-20130104-svn | <http://code.google.com/p/bizmoso/> | Apache License 2.0 | http://www.apache.org/licenses/LICENSE-2.0 | File | Ship | [Copyright © by chatphol.t@gmail.com](mailto:chatphol.t@gmail.com) |
| BlueOxygen Cimande | 2.0rc-sdk | <http://sourceforge.net/projects/cimande/> | Apache License 2.0 | http://www.apache.org/licenses/LICENSE-2.0 | File | Ship | © 2014 - 2015, Meruvian Systems |
| bubble-map |  | <https://www.npmjs.org/package/bubble-map> | MIT License | http://opensource.org/licenses/MIT | Snippet | Ship | Copyright (c) jootse84 |
| c3-angular-sample | 1.2 | <http://github.com/jettro/c3-angular-sample/> | MIT License | http://opensource.org/licenses/MIT | File | Ship | Copyright (c) 2016 Jettro Coenradie |
| codebox7 | trunk-20130330-svn | <http://code.google.com/p/codebox7/> | Apache License 2.0 | http://www.apache.org/licenses/LICENSE-2.0 | Snippet (+ File) | Ship | [Copyright © by JARP87@gmail.com](mailto:JARP87@gmail.com) |
| commons-lang3-3.4.jar | 3.4 | <http://mvnrepository.com/artifact/org.apache.commons/commons-lang3/3.4> | Apache License 2.0 | http://www.apache.org/licenses/LICENSE-2.0 | Component (Dynamic Library) | Ship | Copyright © 2016 The Apache Software Foundation |
| genie-web | 2 | <https://github.com/Netflix/genie> | Apache License 2.0 | http://www.apache.org/licenses/LICENSE-2.0 | File | Ship | Copyright 2015 Netflix, Inc. |
| grails-spring-security-cas | master-20100908 | <http://github.com/grails-plugins/grails-spring-security-cas/> | Apache License 2.0 | http://www.apache.org/licenses/LICENSE-2.0 | File | Ship | Copyright 2006-2015 Burt Beckwith |
| hive-common.jar | 0.8.0 | <http://mvnrepository.com/artifact/org.apache.hive/hive-common/0.8.0> | Apache License 2.0 | http://www.apache.org/licenses/LICENSE-2.0 | Component (Dynamic Library) | Ship | Copyright © 2016 The Apac he Software Foundation |
| j2se-codeserver | trunk-20121206-svn | <http://code.google.com/p/j2se-codeserver/> | BSD 3-clause "New" or "Revised" License | http://opensource.org/licenses/BSD-3-Clause | File | Ship | Copyright © by d925529@gmail.com |
| JBPM Form Modeler Showcase WebApp | 06/04/00 | <http://www.jboss.org/drools/jbpm-form-modeler/jbpm-form-modeler-showcase> | Apache License 2.0 | http://www.apache.org/licenses/LICENSE-2.0 | Snippet (+ File) | Ship | © Copyright 2006-2016, Red Hat, Inc. or third-party contributors |
| Joinfuse | 1_cand_20140820 | <http://github.com/kynetx/Joinfuse/> | MIT License | http://opensource.org/licenses/MIT | File | Ship | Copyright (c) 2013 Kynetx |
| jQuery UI (Combined Library) | 1.11.3 | <http://www.nuget.org/packages/jQuery.UI.Combined> | MIT License | http://opensource.org/licenses/MIT | File | Ship | Copyright 2016 The jQuery Foundation |
| jQuery UI - jquery-ui from code.google.com |  | <http://code.google.com/p/jquery-ui/> | MIT License | http://opensource.org/licenses/MIT | File | Ship | Copyright jQuery Foundation and other contributors, https://jquery.org/ |
| jquery.msgBox.js | 01/01/00 | <https://plugins.jquery.com/msgbox/> | MIT License | http://opensource.org/licenses/MIT | File | Ship | Copyright 2016 The jQuery Foundation. jQuery License |
| jQuery4PHP | master-20130207 | <http://sourceforge.net/projects/jquery4php/> | MIT License | http://opensource.org/licenses/MIT | File | Ship | [© 2010 The jQuery4PHP Project](http://sourceforge.net/projects/jquery4php/) |
| jsdelivr | 1.2 | <http://github.com/jimaek/jsdelivr/> | MIT License | http://opensource.org/licenses/MIT | Snippet (+ File) | Ship | Copyright (c) 2014-2016 Dmitriy Akulov |
| lordofgrid |  | <https://www.npmjs.org/package/lordofgrid> | ISC License | https://opensource.org/licenses/ISC | File | Ship | Copyright (c) Yusuf Zeren |
| Mifos- MicroFinance Open Source | 2.11.0 | <http://sourceforge.net/projects/mifos/> | Apache License 2.0 | http://www.apache.org/licenses/LICENSE-2.0 | File | Ship | (c) Copyright 2014 - Mifos Initiative |
| moment | trunk-20130314-svn | <https://www.npmjs.org/package/moment> | MIT License | http://opensource.org/licenses/MIT | File | Ship | Copyright (c) 2011-2016 Tim Wood, Iskren Chernev, Moment.js contributors |
| msgBoxBackGround.png | master-20140710 | <https://github.com/dotCtor/jQuery.msgBox/tree/master/images> | MIT License | http://opensource.org/licenses/MIT | File | Ship | Copyright (c) 2011-2013 Halil İbrahim Kalyoncu and Oliver Kopp |
| nonglam-fashionshop-dh10dt |  | <http://code.google.com/p/nonglam-fashionshop-dh10dt/> | Apache License 2.0 | http://www.apache.org/licenses/LICENSE-2.0 | Snippet | Ship | Copyright © by thanh100111@gmail.com |
| PFTT2 |  | <http://github.com/OSTC/PFTT2/> | BSD 3-clause "New" or "Revised" License | http://opensource.org/licenses/BSD-3-Clause | File | Ship | Copyright (c) 2012, Microsoft Corporation |
| plotly.js | 1.0.4 | <https://www.npmjs.org/package/plotly.js> | MIT License | http://opensource.org/licenses/MIT | Snippet | Ship | Copyright (c) 2016 Plotly, Inc |
| proyecto6tosvnp | 2.6 | <http://code.google.com/p/proyecto6tosvnp/> | Apache License 2.0 | http://www.apache.org/licenses/LICENSE-2.0 | Snippet | Ship | Copyright © by danielpc01 |
| rails-data-explorer | 1.6 | <http://rubygems.org/gems/rails-data-explorer> | MIT License | http://opensource.org/licenses/MIT | Snippet | Ship | Copyright (c) 2014 Jo Hund |
| Sail.JavaScript | 1.0.0 | <http://www.nuget.org/packages/Sail.JavaScript> | Apache License 2.0 | http://www.apache.org/licenses/LICENSE-2.0 | Snippet | Ship | Copyright 2015 hydra |
| scalaj-collection_2.10-1.6.jar |  | <http://mvnrepository.com/artifact/org.scalaj/scalaj-collection_2.10/1.6> | Apache License 2.0 | http://www.apache.org/licenses/LICENSE-2.0 | Component (Dynamic Library) | Ship | Copyright © org.scalaj |
| sequel-impala |  | <https://rubygems.org/gems/sequel-impala> | MIT License | http://opensource.org/licenses/MIT | File | Ship | Copyright (c) 2015 Jeremy Evans |
| Simple Logging Facade for Java (SLF4J) |  | <http://www.slf4j.org/> | MIT License | http://opensource.org/licenses/MIT | File | Ship | Copyright (c) 2004-2013 QOS.ch |
| The Open For Business Project | trunk-20140322-svn | <http://sourceforge.net/projects/ofbiz/> | MIT License | http://opensource.org/licenses/MIT | File | Ship | Copyright © 2016 The Apache Software Foundation - Apache OFBiz, Apache, the Apache feather logo are trademarks of The Apache Software Foundation. |
| topojson | 2 | <https://www.npmjs.org/package/topojson> | BSD 3-clause "New" or "Revised" License | http://opensource.org/licenses/BSD-3-Clause | File | Ship | Copyright (c) 2012-2016, Michael Bostock |
| vraptor |  | <http://github.com/caelum/vraptor/> | Apache License 2.0 | http://www.apache.org/licenses/LICENSE-2.0 | File | Ship | Copyright (c) 2009 Caelum - www.caelum.com.br/opensource |
| webgme | trunk-20120930-svn | <http://github.com/webgme/webgme/> | MIT License | http://opensource.org/licenses/MIT | File | Ship | Copyright (c) 2012-2016 Vanderbilt University, ISIS |
| wson-application-wsroot | 3 | <http://code.google.com/p/wson-application-wsroot/> | Apache License 2.0 | http://www.apache.org/licenses/LICENSE-2.0 | File | Ship | Copyright © by hangchen222@gmail.com |
| x3dom | 3.2.1 | <http://github.com/x3dom/x3dom/> | MIT License | http://opensource.org/licenses/MIT | Snippet | Ship | Copyright (c) 2009 X3DOM |
| TireBias | 1.6.19 | <http://tirebias.codeplex.com/> | Microsoft Public License | http://opensource.org/licenses/MS-PL | Snippet | Ship | © jittra_yb |

|  |  |  |  |  |  |  |  |
| --- | --- | --- | --- | --- | --- | --- | --- |
| **Unmodified OSS component used and not compiled** | | |  |  |  |  |  |
| **Component** | **Version** | **Home Page** | **License** | **License URL** | **Usage** | **Ship Status** | **Attribution Note** |
| jstl-1.2.jar | 2.1 | <http://mvnrepository.com/artifact/javax.servlet/jstl/1.2> | Common Development and Distribution License 1.0 | http://opensource.org/licenses/CDDL-1.0 | Component (Dynamic Library) | Ship | Copyright 2005 Sun Microsystems |
| liferay-portal |  | <http://github.com/liferay/liferay-portal/> | GNU Lesser General Public License v2.1 or later | http://www.gnu.org/licenses/lgpl-2.1.html | Component (Dynamic Library) | Ship | © 2016 Liferay Inc |
| PHPDevShell | 2 | http://sourceforge.net/projects/phpdevshell/ | GNU Lesser General Public License v2.1 or later | http://www.gnu.org/licenses/lgpl-2.1.html | Component (Dynamic Library) | Ship | Copyright 2009 PHPDevShell.org All rights reserved |

Licenses Texts:

1. Apache License 2.0

| **License** | **Used By** | **Text** |
| --- | --- | --- |
| Apache License 2.0 | Apache Rave, vraptor, Apache Commons Collections, nonglam-fashionshop-dh10dt, Apache-Jakarta Codec, proyecto6tosvnp, Apache Nutch, genie-web, BlueOxygen Cimande, Apache XMLBeans, Mifos- MicroFinance Open Source, grails-spring-security-cas, wson-application-wsroot, Apache Log4j, JBPM Form Modeler Showcase WebApp, scalaj-collection_2.10-1.6.jar, hive-common.jar, Sail.JavaScript, Apache Spark, Apache-Jakarta BeanUtils, codebox7, commons-lang3-3.4.jar, bizmoso | **Apache License** |
|  |  | **Version 2.0, January 2004** |
|  |  |  |
|  |  |  |
|  |  | http://www.apache.org/licenses/ |
|  |  |  |
|  |  | TERMS AND CONDITIONS FOR USE, REPRODUCTION, AND DISTRIBUTION |
|  |  |  |
|  |  | **1. Definitions**. |
|  |  |  |
|  |  | "License" shall mean the terms and conditions for use, reproduction, and distribution as defined by Sections 1 through 9 of this document. |
|  |  |  |
|  |  | "Licensor" shall mean the copyright owner or entity authorized by the copyright owner that is granting the License. |
|  |  |  |
|  |  | "Legal Entity" shall mean the union of the acting entity and all other entities that control, are controlled by, or are under common control with that entity. For the purposes of this definition, "control" means (i) the power, direct or indirect, to cause the direction or management of such entity, whether by contract or otherwise, or (ii) ownership of fifty percent (50%) or more of the outstanding shares, or (iii) beneficial ownership of such entity. |
|  |  |  |
|  |  | "You" (or "Your") shall mean an individual or Legal Entity exercising permissions granted by this License. |
|  |  |  |
|  |  | "Source" form shall mean the preferred form for making modifications, including but not limited to software source code, documentation source, and configuration files. |
|  |  |  |
|  |  | "Object" form shall mean any form resulting from mechanical transformation or translation of a Source form, including but not limited to compiled object code, generated documentation, and conversions to other media types. |
|  |  |  |
|  |  | "Work" shall mean the work of authorship, whether in Source or Object form, made available under the License, as indicated by a copyright notice that is included in or attached to the work (an example is provided in the Appendix below). |
|  |  |  |
|  |  | "Derivative Works" shall mean any work, whether in Source or Object form, that is based on (or derived from) the Work and for which the editorial revisions, annotations, elaborations, or other modifications represent, as a whole, an original work of authorship. For the purposes of this License, Derivative Works shall not include works that remain separable from, or merely link (or bind by name) to the interfaces of, the Work and Derivative Works thereof. |
|  |  |  |
|  |  | "Contribution" shall mean any work of authorship, including the original version of the Work and any modifications or additions to that Work or Derivative Works thereof, that is intentionally submitted to Licensor for inclusion in the Work by the copyright owner or by an individual or Legal Entity authorized to submit on behalf of the copyright owner. For the purposes of this definition, "submitted" means any form of electronic, verbal, or written communication sent to the Licensor or its representatives, including but not limited to communication on electronic mailing lists, source code control systems, and issue tracking systems that are managed by, or on behalf of, the Licensor for the purpose of discussing and improving the Work, but excluding communication that is conspicuously marked or otherwise designated in writing by the copyright owner as "Not a Contribution." |
|  |  |  |
|  |  | "Contributor" shall mean Licensor and any individual or Legal Entity on behalf of whom a Contribution has been received by Licensor and subsequently incorporated within the Work. |
|  |  |  |
|  |  | **2. Grant of Copyright License**. Subject to the terms and conditions of this License, each Contributor hereby grants to You a perpetual, worldwide, non-exclusive, no-charge, royalty-free, irrevocable copyright license to reproduce, prepare Derivative Works of, publicly display, publicly perform, sublicense, and distribute the Work and such Derivative Works in Source or Object form. |
|  |  |  |
|  |  | **3. Grant of Patent License**. Subject to the terms and conditions of this License, each Contributor hereby grants to You a perpetual, worldwide, non-exclusive, no-charge, royalty-free, irrevocable (except as stated in this section) patent license to make, have made, use, offer to sell, sell, import, and otherwise transfer the Work, where such license applies only to those patent claims licensable by such Contributor that are necessarily infringed by their Contribution(s) alone or by combination of their Contribution(s) with the Work to which such Contribution(s) was submitted. If You institute patent litigation against any entity (including a cross-claim or counterclaim in a lawsuit) alleging that the Work or a Contribution incorporated within the Work constitutes direct or contributory patent infringement, then any patent licenses granted to You under this License for that Work shall terminate as of the date such litigation is filed. |
|  |  |  |
|  |  | **4. Redistribution**. You may reproduce and distribute copies of the Work or Derivative Works thereof in any medium, with or without modifications, and in Source or Object form, provided that You meet the following conditions: |
|  |  |  |
|  |  | a. You must give any other recipients of the Work or Derivative Works a copy of this License; and |
|  |  |  |
|  |  | b. You must cause any modified files to carry prominent notices stating that You changed the files; and |
|  |  |  |
|  |  | c. You must retain, in the Source form of any Derivative Works that You distribute, all copyright, patent, trademark, and attribution notices from the Source form of the Work, excluding those notices that do not pertain to any part of the Derivative Works; and |
|  |  |  |
|  |  | d. If the Work includes a "NOTICE" text file as part of its distribution, then any Derivative Works that You distribute must include a readable copy of the attribution notices contained within such NOTICE file, excluding those notices that do not pertain to any part of the Derivative Works, in at least one of the following places: within a NOTICE text file distributed as part of the Derivative Works; within the Source form or documentation, if provided along with the Derivative Works; or, within a display generated by the Derivative Works, if and wherever such third-party notices normally appear. The contents of the NOTICE file are for informational purposes only and do not modify the License. You may add Your own attribution notices within Derivative Works that You distribute, alongside or as an addendum to the NOTICE text from the Work, provided that such additional attribution notices cannot be construed as modifying the License. |
|  |  |  |
|  |  | You may add Your own copyright statement to Your modifications and may provide additional or different license terms and conditions for use, reproduction, or distribution of Your modifications, or for any such Derivative Works as a whole, provided Your use, reproduction, and distribution of the Work otherwise complies with the conditions stated in this License. |
|  |  |  |
|  |  | **5. Submission of Contributions**. Unless You explicitly state otherwise, any Contribution intentionally submitted for inclusion in the Work by You to the Licensor shall be under the terms and conditions of this License, without any additional terms or conditions. Notwithstanding the above, nothing herein shall supersede or modify the terms of any separate license agreement you may have executed with Licensor regarding such Contributions. |
|  |  |  |
|  |  | **6. Trademarks**. This License does not grant permission to use the trade names, trademarks, service marks, or product names of the Licensor, except as required for reasonable and customary use in describing the origin of the Work and reproducing the content of the NOTICE file. |
|  |  |  |
|  |  | **7. Disclaimer of Warranty**. Unless required by applicable law or agreed to in writing, Licensor provides the Work (and each Contributor provides its Contributions) on an "AS IS" BASIS, WITHOUT WARRANTIES OR CONDITIONS OF ANY KIND, either express or implied, including, without limitation, any warranties or conditions of TITLE, NON-INFRINGEMENT, MERCHANTABILITY, or FITNESS FOR A PARTICULAR PURPOSE. You are solely responsible for determining the appropriateness of using or redistributing the Work and assume any risks associated with Your exercise of permissions under this License. |
|  |  |  |
|  |  | **8. Limitation of Liability**. In no event and under no legal theory, whether in tort (including negligence), contract, or otherwise, unless required by applicable law (such as deliberate and grossly negligent acts) or agreed to in writing, shall any Contributor be liable to You for damages, including any direct, indirect, special, incidental, or consequential damages of any character arising as a result of this License or out of the use or inability to use the Work (including but not limited to damages for loss of goodwill, work stoppage, computer failure or malfunction, or any and all other commercial damages or losses), even if such Contributor has been advised of the possibility of such damages. |
|  |  |  |
|  |  | **9. Accepting Warranty or Additional Liability**. While redistributing the Work or Derivative Works thereof, You may choose to offer, and charge a fee for, acceptance of support, warranty, indemnity, or other liability obligations and/or rights consistent with this License. However, in accepting such obligations, You may act only on Your own behalf and on Your sole responsibility, not on behalf of any other Contributor, and only if You agree to indemnify, defend, and hold each Contributor harmless for any liability incurred by, or claims asserted against, such Contributor by reason of your accepting any such warranty or additional liability. |
|  |  |  |
|  |  | END OF TERMS AND CONDITIONS |
|  |  |  |
|  |  | **APPENDIX: How to apply the Apache License to your work** |
|  |  |  |
|  |  | To apply the Apache License to your work, attach the following boilerplate notice, with the fields enclosed by brackets "[]" replaced with your own identifying information. (Don't include the brackets!) The text should be enclosed in the appropriate comment syntax for the file format. We also recommend that a file or class name and description of purpose be included on the same "printed page" as the copyright notice for easier identification within third-party archives. |
|  |  |  |
|  |  | Copyright [yyyy] [name of copyright owner] Licensed under the Apache License, Version 2.0 (the "License"); you may not use this file except in compliance with the License. You may obtain a copy of the License at http://www.apache.org/licenses/LICENSE-2.0 Unless required by applicable law or agreed to in writing, software distributed under the License is distributed on an "AS IS" BASIS, WITHOUT WARRANTIES OR CONDITIONS OF ANY KIND, either express or implied. See the License for the specific language governing permissions and limitations under the License. |
|  |  |  |

1. MIT License

| **License** | **Used By** | **Text** |
| --- | --- | --- |
| MIT License | jquery.msgBox.js, The Open For Business Project, jsdelivr, webgme, jQuery UI (Combined Library), c3-angular-sample, bubble-map, plotly.js, jQuery4PHP, jQuery UI - jquery-ui from code.google.com, rails-data-explorer, moment, x3dom, sequel-impala, Simple Logging Facade for Java (SLF4J), Joinfuse, msgBoxBackGround.png | **The MIT License** |
|  |  |  |
|  |  | Copyright (c) <year> <copyright holders> |
|  |  |  |
|  |  | Permission is hereby granted, free of charge, to any person obtaining a copy of this software and associated documentation files (the "Software"), to deal in the Software without restriction, including without limitation the rights to use, copy, modify, merge, publish, distribute, sublicense, and/or sell copies of the Software, and to permit persons to whom the Software is furnished to do so, subject to the following conditions: |
|  |  |  |
|  |  | The above copyright notice and this permission notice shall be included in all copies or substantial portions of the Software. |
|  |  |  |
|  |  | THE SOFTWARE IS PROVIDED "AS IS", WITHOUT WARRANTY OF ANY KIND, EXPRESS OR IMPLIED, INCLUDING BUT NOT LIMITED TO THE WARRANTIES OF MERCHANTABILITY, FITNESS FOR A PARTICULAR PURPOSE AND NONINFRINGEMENT. IN NO EVENT SHALL THE AUTHORS OR COPYRIGHT HOLDERS BE LIABLE FOR ANY CLAIM, DAMAGES OR OTHER LIABILITY, WHETHER IN AN ACTION OF CONTRACT, TORT OR OTHERWISE, ARISING FROM, OUT OF OR IN CONNECTION WITH THE SOFTWARE OR THE USE OR OTHER DEALINGS IN THE SOFTWARE. |

1. ISC License

| **License** | **Used By** | **Text** |
| --- | --- | --- |
| ISC License | lordofgrid | **ISC License (ISCL)** |
|  |  |  |
|  |  | Copyright (c) 4-digit year, Company or Person's Name |
|  |  |  |
|  |  | Permission to use, copy, modify, and/or distribute this software for any purpose with or without fee is hereby granted, provided that the above copyright notice and this permission notice appear in all copies. |
|  |  |  |
|  |  | THE SOFTWARE IS PROVIDED "AS IS" AND THE AUTHOR DISCLAIMS ALL WARRANTIES WITH REGARD TO THIS SOFTWARE INCLUDING ALL IMPLIED WARRANTIES OF MERCHANTABILITY AND FITNESS. IN NO EVENT SHALL THE AUTHOR BE LIABLE FOR ANY SPECIAL, DIRECT, INDIRECT, OR CONSEQUENTIAL DAMAGES OR ANY DAMAGES WHATSOEVER RESULTING FROM LOSS OF USE, DATA OR PROFITS, WHETHER IN AN ACTION OF CONTRACT, NEGLIGENCE OR OTHER TORTIOUS ACTION, ARISING OUT OF OR IN CONNECTION WITH THE USE OR PERFORMANCE OF THIS SOFTWARE. |

1. BSD 3-clause "New" or "Revised" License

| **License** | **Used By** | **Text** |
| --- | --- | --- |
| BSD 3-clause "New" or "Revised" License | topojson, j2se-codeserver, PFTT2 |  |
|  |  | Copyright (c) <YEAR>, <OWNER> |
|  |  | All rights reserved. |
|  |  |  |
|  |  | Redistribution and use in source and binary forms, with or without modification, are permitted provided that the following conditions are met: |
|  |  |  |
|  |  | Redistributions of source code must retain the above copyright notice, this list of conditions and the following disclaimer. |
|  |  | Redistributions in binary form must reproduce the above copyright notice, this list of conditions and the following disclaimer in the documentation and/or other materials provided with the distribution. |
|  |  | Neither the name of the <ORGANIZATION> nor the names of its contributors may be used to endorse or promote products derived from this software without specific prior written permission. |
|  |  |  |
|  |  |  |
|  |  | THIS SOFTWARE IS PROVIDED BY THE COPYRIGHT HOLDERS AND CONTRIBUTORS "AS IS" AND ANY EXPRESS OR IMPLIED WARRANTIES, INCLUDING, BUT NOT LIMITED TO, THE IMPLIED WARRANTIES OF MERCHANTABILITY AND FITNESS FOR A PARTICULAR PURPOSE ARE DISCLAIMED. IN NO EVENT SHALL THE COPYRIGHT OWNER OR CONTRIBUTORS BE LIABLE FOR ANY DIRECT, INDIRECT, INCIDENTAL, SPECIAL, EXEMPLARY, OR CONSEQUENTIAL DAMAGES (INCLUDING, BUT NOT LIMITED TO, PROCUREMENT OF SUBSTITUTE GOODS OR SERVICES; LOSS OF USE, DATA, OR PROFITS; OR BUSINESS INTERRUPTION) HOWEVER CAUSED AND ON ANY THEORY OF LIABILITY, WHETHER IN CONTRACT, STRICT LIABILITY, OR TORT (INCLUDING NEGLIGENCE OR OTHERWISE) ARISING IN ANY WAY OUT OF THE USE OF THIS SOFTWARE, EVEN IF ADVISED OF THE POSSIBILITY OF SUCH DAMAGE. |
|  |  |  |

1. Microsoft Public License

| **License** | **Used By** | **Text** |
| --- | --- | --- |
| Microsoft Public License | TireBias | **Microsoft Public License (Ms-PL)** |
|  |  |  |
|  |  | This license governs use of the accompanying software. If you use the software, you accept this license. If you do not accept the license, do not use the software. |
|  |  |  |
|  |  | 1. Definitions |
|  |  | The terms "reproduce," "reproduction," "derivative works," and "distribution" have the same meaning here as under U.S. copyright law. |
|  |  | A "contribution" is the original software, or any additions or changes to the software. |
|  |  | A "contributor" is any person that distributes its contribution under this license. |
|  |  | "Licensed patents" are a contributor's patent claims that read directly on its contribution. |
|  |  |  |
|  |  | 2. Grant of Rights |
|  |  |  |
|  |  |  |
|  |  | A. Copyright Grant- Subject to the terms of this license, including the license conditions and limitations in section 3, each contributor grants you a non-exclusive, worldwide, royalty-free copyright license to reproduce its contribution, prepare derivative works of its contribution, and distribute its contribution or any derivative works that you create. |
|  |  |  |
|  |  | B. Patent Grant- Subject to the terms of this license, including the license conditions and limitations in section 3, each contributor grants you a non-exclusive, worldwide, royalty-free license under its licensed patents to make, have made, use, sell, offer for sale, import, and/or otherwise dispose of its contribution in the software or derivative works of the contribution in the software. |
|  |  |  |
|  |  | 3. Conditions and Limitations |
|  |  |  |
|  |  |  |
|  |  | A. No Trademark License- This license does not grant you rights to use any contributors' name, logo, or trademarks. |
|  |  |  |
|  |  | B. If you bring a patent claim against any contributor over patents that you claim are infringed by the software, your patent license from such contributor to the software ends automatically. |
|  |  |  |
|  |  | C. If you distribute any portion of the software, you must retain all copyright, patent, trademark, and attribution notices that are present in the software. |
|  |  |  |
|  |  | D. If you distribute any portion of the software in source code form, you may do so only under this license by including a complete copy of this license with your distribution. If you distribute any portion of the software in compiled or object code form, you may only do so under a license that complies with this license. |
|  |  |  |
|  |  | E. The software is licensed "as-is." You bear the risk of using it. The contributors give no express warranties, guarantees or conditions. You may have additional consumer rights under your local laws which this license cannot change. To the extent permitted under your local laws, the contributors exclude the implied warranties of merchantability, fitness for a particular purpose and non-infringement. |

1. GNU Lesser General Public License v2.1 or later

| **License** | **Used By** | **Text** |
| --- | --- | --- |
| GNU Lesser General Public License v2.1 or later | liferay-portal, PHPDevShell | **GNU Lesser General Public License** |
|  |  |  |
|  |  | Version 2.1, February 1999 |
|  |  |  |
|  |  | Copyright (C) 1991, 1999 Free Software Foundation, Inc. |
|  |  |  |
|  |  | 59 Temple Place, Suite 330, Boston, MA 02111-1307 USA |
|  |  |  |
|  |  | Everyone is permitted to copy and distribute verbatim copies |
|  |  |  |
|  |  | of this license document, but changing it is not allowed. |
|  |  |  |
|  |  | [This is the first released version of the Lesser GPL. It also counts |
|  |  |  |
|  |  | as the successor of the GNU Library Public License, version 2, hence |
|  |  |  |
|  |  | the version number 2.1.] |
|  |  |  |
|  |  |  |
|  |  | **Preamble** |
|  |  |  |
|  |  | The licenses for most software are designed to take away your freedom to share and change it. By contrast, the GNU General Public Licenses are intended to guarantee your freedom to share and change free software--to make sure the software is free for all its users. |
|  |  |  |
|  |  | This license, the Lesser General Public License, applies to some specially designated software packages--typically libraries--of the Free Software Foundation and other authors who decide to use it. You can use it too, but we suggest you first think carefully about whether this license or the ordinary General Public License is the better strategy to use in any particular case, based on the explanations below. |
|  |  |  |
|  |  | When we speak of free software, we are referring to freedom of use, not price. Our General Public Licenses are designed to make sure that you have the freedom to distribute copies of free software (and charge for this service if you wish); that you receive source code or can get it if you want it; that you can change the software and use pieces of it in new free programs; and that you are informed that you can do these things. |
|  |  |  |
|  |  | To protect your rights, we need to make restrictions that forbid distributors to deny you these rights or to ask you to surrender these rights. These restrictions translate to certain responsibilities for you if you distribute copies of the library or if you modify it. |
|  |  |  |
|  |  | For example, if you distribute copies of the library, whether gratis or for a fee, you must give the recipients all the rights that we gave you. You must make sure that they, too, receive or can get the source code. If you link other code with the library, you must provide complete object files to the recipients, so that they can relink them with the library after making changes to the library and recompiling it. And you must show them these terms so they know their rights. |
|  |  |  |
|  |  | We protect your rights with a two-step method: (1) we copyright the library, and (2) we offer you this license, which gives you legal permission to copy, distribute and/or modify the library. |
|  |  |  |
|  |  | To protect each distributor, we want to make it very clear that there is no warranty for the free library. Also, if the library is modified by someone else and passed on, the recipients should know that what they have is not the original version, so that the original author's reputation will not be affected by problems that might be introduced by others. |
|  |  |  |
|  |  | Finally, software patents pose a constant threat to the existence of any free program. We wish to make sure that a company cannot effectively restrict the users of a free program by obtaining a restrictive license from a patent holder. Therefore, we insist that any patent license obtained for a version of the library must be consistent with the full freedom of use specified in this license. |
|  |  |  |
|  |  | Most GNU software, including some libraries, is covered by the ordinary GNU General Public License. This license, the GNU Lesser General Public License, applies to certain designated libraries, and is quite different from the ordinary General Public License. We use this license for certain libraries in order to permit linking those libraries into non-free programs. |
|  |  |  |
|  |  | When a program is linked with a library, whether statically or using a shared library, the combination of the two is legally speaking a combined work, a derivative of the original library. The ordinary General Public License therefore permits such linking only if the entire combination fits its criteria of freedom. The Lesser General Public License permits more lax criteria for linking other code with the library. |
|  |  |  |
|  |  | We call this license the "Lesser" General Public License because it does Less to protect the user's freedom than the ordinary General Public License. It also provides other free software developers Less of an advantage over competing non-free programs. These disadvantages are the reason we use the ordinary General Public License for many libraries. However, the Lesser license provides advantages in certain special circumstances. |
|  |  |  |
|  |  | For example, on rare occasions, there may be a special need to encourage the widest possible use of a certain library, so that it becomes a de-facto standard. To achieve this, non-free programs must be allowed to use the library. A more frequent case is that a free library does the same job as widely used non-free libraries. In this case, there is little to gain by limiting the free library to free software only, so we use the Lesser General Public License. |
|  |  |  |
|  |  | In other cases, permission to use a particular library in non-free programs enables a greater number of people to use a large body of free software. For example, permission to use the GNU C Library in non-free programs enables many more people to use the whole GNU operating system, as well as its variant, the GNU/Linux operating system. |
|  |  |  |
|  |  | Although the Lesser General Public License is Less protective of the users' freedom, it does ensure that the user of a program that is linked with the Library has the freedom and the wherewithal to run that program using a modified version of the Library. |
|  |  |  |
|  |  | The precise terms and conditions for copying, distribution and modification follow. Pay close attention to the difference between a "work based on the library" and a "work that uses the library". The former contains code derived from the library, whereas the latter must be combined with the library in order to run. |
|  |  |  |
|  |  | **TERMS AND CONDITIONS FOR COPYING, DISTRIBUTION AND MODIFICATION** |
|  |  |  |
|  |  | **0.** This License Agreement applies to any software library or other program which contains a notice placed by the copyright holder or other authorized party saying it may be distributed under the terms of this Lesser General Public License (also called "this License"). Each licensee is addressed as "you". |
|  |  |  |
|  |  | A "library" means a collection of software functions and/or data prepared so as to be conveniently linked with application programs (which use some of those functions and data) to form executables. |
|  |  |  |
|  |  | The "Library", below, refers to any such software library or work which has been distributed under these terms. A "work based on the Library" means either the Library or any derivative work under copyright law: that is to say, a work containing the Library or a portion of it, either verbatim or with modifications and/or translated straightforwardly into another language. (Hereinafter, translation is included without limitation in the term "modification".) |
|  |  |  |
|  |  | "Source code" for a work means the preferred form of the work for making modifications to it. For a library, complete source code means all the source code for all modules it contains, plus any associated interface definition files, plus the scripts used to control compilation and installation of the library. |
|  |  |  |
|  |  | Activities other than copying, distribution and modification are not covered by this License; they are outside its scope. The act of running a program using the Library is not restricted, and output from such a program is covered only if its contents constitute a work based on the Library (independent of the use of the Library in a tool for writing it). Whether that is true depends on what the Library does and what the program that uses the Library does. |
|  |  |  |
|  |  | **1.** You may copy and distribute verbatim copies of the Library's complete source code as you receive it, in any medium, provided that you conspicuously and appropriately publish on each copy an appropriate copyright notice and disclaimer of warranty; keep intact all the notices that refer to this License and to the absence of any warranty; and distribute a copy of this License along with the Library. |
|  |  |  |
|  |  | You may charge a fee for the physical act of transferring a copy, and you may at your option offer warranty protection in exchange for a fee. |
|  |  |  |
|  |  | **2.** You may modify your copy or copies of the Library or any portion of it, thus forming a work based on the Library, and copy and distribute such modifications or work under the terms of Section 1 above, provided that you also meet all of these conditions: |
|  |  |  |
|  |  | a) The modified work must itself be a software library. |
|  |  |  |
|  |  | b) You must cause the files modified to carry prominent notices stating that you changed the files and the date of any change. |
|  |  |  |
|  |  | c) You must cause the whole of the work to be licensed at no charge to all third parties under the terms of this License. |
|  |  |  |
|  |  | d) If a facility in the modified Library refers to a function or a table of data to be supplied by an application program that uses the facility, other than as an argument passed when the facility is invoked, then you must make a good faith effort to ensure that, in the event an application does not supply such function or table, the facility still operates, and performs whatever part of its purpose remains meaningful. |
|  |  |  |
|  |  | (For example, a function in a library to compute square roots has a purpose that is entirely well-defined independent of the application. Therefore, Subsection 2d requires that any application-supplied function or table used by this function must be optional: if the application does not supply it, the square root function must still compute square roots.) |
|  |  |  |
|  |  | These requirements apply to the modified work as a whole. If identifiable sections of that work are not derived from the Library, and can be reasonably considered independent and separate works in themselves, then this License, and its terms, do not apply to those sections when you distribute them as separate works. But when you distribute the same sections as part of a whole which is a work based on the Library, the distribution of the whole must be on the terms of this License, whose permissions for other licensees extend to the entire whole, and thus to each and every part regardless of who wrote it. |
|  |  |  |
|  |  | Thus, it is not the intent of this section to claim rights or contest your rights to work written entirely by you; rather, the intent is to exercise the right to control the distribution of derivative or collective works based on the Library. |
|  |  |  |
|  |  | In addition, mere aggregation of another work not based on the Library with the Library (or with a work based on the Library) on a volume of a storage or distribution medium does not bring the other work under the scope of this License. |
|  |  |  |
|  |  | 3. You may opt to apply the terms of the ordinary GNU General Public License instead of this License to a given copy of the Library. To do this, you must alter all the notices that refer to this License, so that they refer to the ordinary GNU General Public License, version 2, instead of to this License. (If a newer version than version 2 of the ordinary GNU General Public License has appeared, then you can specify that version instead if you wish.) Do not make any other change in these notices. |
|  |  |  |
|  |  | Once this change is made in a given copy, it is irreversible for that copy, so the ordinary GNU General Public License applies to all subsequent copies and derivative works made from that copy. |
|  |  |  |
|  |  | This option is useful when you wish to copy part of the code of the Library into a program that is not a library. |
|  |  |  |
|  |  | 4. You may copy and distribute the Library (or a portion or derivative of it, under Section 2) in object code or executable form under the terms of Sections 1 and 2 above provided that you accompany it with the complete corresponding machine-readable source code, which must be distributed under the terms of Sections 1 and 2 above on a medium customarily used for software interchange. |
|  |  |  |
|  |  | If distribution of object code is made by offering access to copy from a designated place, then offering equivalent access to copy the source code from the same place satisfies the requirement to distribute the source code, even though third parties are not compelled to copy the source along with the object code. |
|  |  |  |
|  |  | 5. A program that contains no derivative of any portion of the Library, but is designed to work with the Library by being compiled or linked with it, is called a "work that uses the Library". Such a work, in isolation, is not a derivative work of the Library, and therefore falls outside the scope of this License. |
|  |  |  |
|  |  | However, linking a "work that uses the Library" with the Library creates an executable that is a derivative of the Library (because it contains portions of the Library), rather than a "work that uses the library". The executable is therefore covered by this License. Section 6 states terms for distribution of such executables. |
|  |  |  |
|  |  | When a "work that uses the Library" uses material from a header file that is part of the Library, the object code for the work may be a derivative work of the Library even though the source code is not. Whether this is true is especially significant if the work can be linked without the Library, or if the work is itself a library. The threshold for this to be true is not precisely defined by law. |
|  |  |  |
|  |  | If such an object file uses only numerical parameters, data structure layouts and accessors, and small macros and small inline functions (ten lines or less in length), then the use of the object file is unrestricted, regardless of whether it is legally a derivative work. (Executables containing this object code plus portions of the Library will still fall under Section 6.) |
|  |  |  |
|  |  | Otherwise, if the work is a derivative of the Library, you may distribute the object code for the work under the terms of Section 6. Any executables containing that work also fall under Section 6, whether or not they are linked directly with the Library itself. |
|  |  |  |
|  |  | 6. As an exception to the Sections above, you may also combine or link a "work that uses the Library" with the Library to produce a work containing portions of the Library, and distribute that work under terms of your choice, provided that the terms permit modification of the work for the customer's own use and reverse engineering for debugging such modifications. |
|  |  |  |
|  |  | You must give prominent notice with each copy of the work that the Library is used in it and that the Library and its use are covered by this License. You must supply a copy of this License. If the work during execution displays copyright notices, you must include the copyright notice for the Library among them, as well as a reference directing the user to the copy of this License. Also, you must do one of these things: |
|  |  |  |
|  |  | a) Accompany the work with the complete corresponding machine-readable source code for the Library including whatever changes were used in the work (which must be distributed under Sections 1 and 2 above); and, if the work is an executable linked with the Library, with the complete machine-readable "work that uses the Library", as object code and/or source code, so that the user can modify the Library and then relink to produce a modified executable containing the modified Library. (It is understood that the user who changes the contents of definitions files in the Library will not necessarily be able to recompile the application to use the modified definitions.) |
|  |  |  |
|  |  | b) Use a suitable shared library mechanism for linking with the Library. A suitable mechanism is one that (1) uses at run time a copy of the library already present on the user's computer system, rather than copying library functions into the executable, and (2) will operate properly with a modified version of the library, if the user installs one, as long as the modified version is interface-compatible with the version that the work was made with. |
|  |  |  |
|  |  | c) Accompany the work with a written offer, valid for at least three years, to give the same user the materials specified in Subsection 6a, above, for a charge no more than the cost of performing this distribution. |
|  |  |  |
|  |  | d) If distribution of the work is made by offering access to copy from a designated place, offer equivalent access to copy the above specified materials from the same place. |
|  |  |  |
|  |  | e) Verify that the user has already received a copy of these materials or that you have already sent this user a copy. |
|  |  |  |
|  |  | For an executable, the required form of the "work that uses the Library" must include any data and utility programs needed for reproducing the executable from it. However, as a special exception, the materials to be distributed need not include anything that is normally distributed (in either source or binary form) with the major components (compiler, kernel, and so on) of the operating system on which the executable runs, unless that component itself accompanies the executable. |
|  |  |  |
|  |  | It may happen that this requirement contradicts the license restrictions of other proprietary libraries that do not normally accompany the operating system. Such a contradiction means you cannot use both them and the Library together in an executable that you distribute. |
|  |  |  |
|  |  | 7. You may place library facilities that are a work based on the Library side-by-side in a single library together with other library facilities not covered by this License, and distribute such a combined library, provided that the separate distribution of the work based on the Library and of the other library facilities is otherwise permitted, and provided that you do these two things: |
|  |  |  |
|  |  | a) Accompany the combined library with a copy of the same work based on the Library, uncombined with any other library facilities. This must be distributed under the terms of the Sections above. |
|  |  |  |
|  |  | b) Give prominent notice with the combined library of the fact that part of it is a work based on the Library, and explaining where to find the accompanying uncombined form of the same work. |
|  |  |  |
|  |  | 8. You may not copy, modify, sublicense, link with, or distribute the Library except as expressly provided under this License. Any attempt otherwise to copy, modify, sublicense, link with, or distribute the Library is void, and will automatically terminate your rights under this License. However, parties who have received copies, or rights, from you under this License will not have their licenses terminated so long as such parties remain in full compliance. |
|  |  |  |
|  |  | 9. You are not required to accept this License, since you have not signed it. However, nothing else grants you permission to modify or distribute the Library or its derivative works. These actions are prohibited by law if you do not accept this License. Therefore, by modifying or distributing the Library (or any work based on the Library), you indicate your acceptance of this License to do so, and all its terms and conditions for copying, distributing or modifying the Library or works based on it. |
|  |  |  |
|  |  | 10. Each time you redistribute the Library (or any work based on the Library), the recipient automatically receives a license from the original licensor to copy, distribute, link with or modify the Library subject to these terms and conditions. You may not impose any further restrictions on the recipients' exercise of the rights granted herein. You are not responsible for enforcing compliance by third parties with this License. |
|  |  |  |
|  |  | 11. If, as a consequence of a court judgment or allegation of patent infringement or for any other reason (not limited to patent issues), conditions are imposed on you (whether by court order, agreement or otherwise) that contradict the conditions of this License, they do not excuse you from the conditions of this License. If you cannot distribute so as to satisfy simultaneously your obligations under this License and any other pertinent obligations, then as a consequence you may not distribute the Library at all. For example, if a patent license would not permit royalty-free redistribution of the Library by all those who receive copies directly or indirectly through you, then the only way you could satisfy both it and this License would be to refrain entirely from distribution of the Library. |
|  |  |  |
|  |  | If any portion of this section is held invalid or unenforceable under any particular circumstance, the balance of the section is intended to apply, and the section as a whole is intended to apply in other circumstances. |
|  |  |  |
|  |  | It is not the purpose of this section to induce you to infringe any patents or other property right claims or to contest validity of any such claims; this section has the sole purpose of protecting the integrity of the free software distribution system which is implemented by public license practices. Many people have made generous contributions to the wide range of software distributed through that system in reliance on consistent application of that system; it is up to the author/donor to decide if he or she is willing to distribute software through any other system and a licensee cannot impose that choice. |
|  |  |  |
|  |  | This section is intended to make thoroughly clear what is believed to be a consequence of the rest of this License. |
|  |  |  |
|  |  | 12. If the distribution and/or use of the Library is restricted in certain countries either by patents or by copyrighted interfaces, the original copyright holder who places the Library under this License may add an explicit geographical distribution limitation excluding those countries, so that distribution is permitted only in or among countries not thus excluded. In such case, this License incorporates the limitation as if written in the body of this License. |
|  |  |  |
|  |  | 13. The Free Software Foundation may publish revised and/or new versions of the Lesser General Public License from time to time. Such new versions will be similar in spirit to the present version, but may differ in detail to address new problems or concerns. |
|  |  |  |
|  |  | Each version is given a distinguishing version number. If the Library specifies a version number of this License which applies to it and "any later version", you have the option of following the terms and conditions either of that version or of any later version published by the Free Software Foundation. If the Library does not specify a license version number, you may choose any version ever published by the Free Software Foundation. |
|  |  |  |
|  |  | 14. If you wish to incorporate parts of the Library into other free programs whose distribution conditions are incompatible with these, write to the author to ask for permission. For software which is copyrighted by the Free Software Foundation, write to the Free Software Foundation; we sometimes make exceptions for this. Our decision will be guided by the two goals of preserving the free status of all derivatives of our free software and of promoting the sharing and reuse of software generally. |
|  |  |  |
|  |  | NO WARRANTY |
|  |  |  |
|  |  | 15. BECAUSE THE LIBRARY IS LICENSED FREE OF CHARGE, THERE IS NO WARRANTY FOR THE LIBRARY, TO THE EXTENT PERMITTED BY APPLICABLE LAW. EXCEPT WHEN OTHERWISE STATED IN WRITING THE COPYRIGHT HOLDERS AND/OR OTHER PARTIES PROVIDE THE LIBRARY "AS IS" WITHOUT WARRANTY OF ANY KIND, EITHER EXPRESSED OR IMPLIED, INCLUDING, BUT NOT LIMITED TO, THE IMPLIED WARRANTIES OF MERCHANTABILITY AND FITNESS FOR A PARTICULAR PURPOSE. THE ENTIRE RISK AS TO THE QUALITY AND PERFORMANCE OF THE LIBRARY IS WITH YOU. SHOULD THE LIBRARY PROVE DEFECTIVE, YOU ASSUME THE COST OF ALL NECESSARY SERVICING, REPAIR OR CORRECTION. |
|  |  |  |
|  |  | 16. IN NO EVENT UNLESS REQUIRED BY APPLICABLE LAW OR AGREED TO IN WRITING WILL ANY COPYRIGHT HOLDER, OR ANY OTHER PARTY WHO MAY MODIFY AND/OR REDISTRIBUTE THE LIBRARY AS PERMITTED ABOVE, BE LIABLE TO YOU FOR DAMAGES, INCLUDING ANY GENERAL, SPECIAL, INCIDENTAL OR CONSEQUENTIAL DAMAGES ARISING OUT OF THE USE OR INABILITY TO USE THE LIBRARY (INCLUDING BUT NOT LIMITED TO LOSS OF DATA OR DATA BEING RENDERED INACCURATE OR LOSSES SUSTAINED BY YOU OR THIRD PARTIES OR A FAILURE OF THE LIBRARY TO OPERATE WITH ANY OTHER SOFTWARE), EVEN IF SUCH HOLDER OR OTHER PARTY HAS BEEN ADVISED OF THE POSSIBILITY OF SUCH DAMAGES. |
|  |  |  |
|  |  | **END OF TERMS AND CONDITIONS** |
|  |  |  |
|  |  |  |
|  |  | **How to Apply These Terms to Your New Libraries** |
|  |  |  |
|  |  | If you develop a new library, and you want it to be of the greatest possible use to the public, we recommend making it free software that everyone can redistribute and change. You can do so by permitting redistribution under these terms (or, alternatively, under the terms of the ordinary General Public License). |
|  |  |  |
|  |  | To apply these terms, attach the following notices to the library. It is safest to attach them to the start of each source file to most effectively convey the exclusion of warranty; and each file should have at least the "copyright" line and a pointer to where the full notice is found. |
|  |  |  |
|  |  | one line to give the library's name and an idea of what it does. |
|  |  |  |
|  |  | Copyright (C) year name of author |
|  |  |  |
|  |  | This library is free software; you can redistribute it and/or |
|  |  |  |
|  |  | modify it under the terms of the GNU Lesser General Public |
|  |  |  |
|  |  | License as published by the Free Software Foundation; either |
|  |  |  |
|  |  | version 2.1 of the License, or (at your option) any later version. |
|  |  |  |
|  |  | This library is distributed in the hope that it will be useful, |
|  |  |  |
|  |  | but WITHOUT ANY WARRANTY; without even the implied warranty of |
|  |  |  |
|  |  | MERCHANTABILITY or FITNESS FOR A PARTICULAR PURPOSE. See the GNU |
|  |  |  |
|  |  | Lesser General Public License for more details. |
|  |  |  |
|  |  | You should have received a copy of the GNU Lesser General Public |
|  |  |  |
|  |  | License along with this library; if not, write to the Free Software |
|  |  |  |
|  |  | Foundation, Inc., 59 Temple Place, Suite 330, Boston, MA 02111-1307 USA |
|  |  |  |
|  |  | Also add information on how to contact you by electronic and paper mail. |
|  |  |  |
|  |  | You should also get your employer (if you work as a programmer) or your school, if any, to sign a "copyright disclaimer" for the library, if necessary. Here is a sample; alter the names: |
|  |  |  |
|  |  | Yoyodyne, Inc., hereby disclaims all copyright interest in |
|  |  |  |
|  |  | the library `Frob' (a library for tweaking knobs) written |
|  |  |  |
|  |  | by James Random Hacker. |
|  |  |  |
|  |  | signature of Ty Coon, 1 April 1990 |
|  |  |  |
|  |  | Ty Coon, President of Vice |
|  |  |  |
|  |  | That's all there is to it! |

1. Common Development and Distribution License 1.0

| **License** | **Used By** | **Text** |
| --- | --- | --- |
| Common Development and Distribution License 1.0 | jstl-1.2.jar | **COMMON DEVELOPMENT AND DISTRIBUTION LICENSE (CDDL) Version 1.0** |
|  |  |  |
|  |  | 1. Definitions. |
|  |  |  |
|  |  | 1.1. "Contributor" means each individual or entity that creates or contributes to the creation of Modifications. |
|  |  |  |
|  |  | 1.2. "Contributor Version" means the combination of the Original Software, prior Modifications used by a Contributor (if any), and the Modifications made by that particular Contributor. |
|  |  |  |
|  |  | 1.3. "Covered Software" means (a) the Original Software, or (b) Modifications, or (c) the combination of files containing Original Software with files containing Modifications, in each case including portions thereof. |
|  |  |  |
|  |  | 1.4. "Executable" means the Covered Software in any form other than Source Code. |
|  |  |  |
|  |  | 1.5. "Initial Developer" means the individual or entity that first makes Original Software available under this License. |
|  |  |  |
|  |  | 1.6. "Larger Work" means a work which combines Covered Software or portions thereof with code not governed by the terms of this License. |
|  |  |  |
|  |  | 1.7. "License" means this document. |
|  |  |  |
|  |  | 1.8. "Licensable" means having the right to grant, to the maximum extent possible, whether at the time of the initial grant or subsequently acquired, any and all of the rights conveyed herein. |
|  |  |  |
|  |  | 1.9. "Modifications" means the Source Code and Executable form of any of the following: |
|  |  |  |
|  |  | A. Any file that results from an addition to, deletion from or modification of the contents of a file containing Original Software or previous Modifications; |
|  |  |  |
|  |  | B. Any new file that contains any part of the Original Software or previous Modification; or |
|  |  |  |
|  |  | C. Any new file that is contributed or otherwise made available under the terms of this License. |
|  |  |  |
|  |  | 1.10. "Original Software" means the Source Code and Executable form of computer software code that is originally released under this License. |
|  |  |  |
|  |  | 1.11. "Patent Claims" means any patent claim(s), now owned or hereafter acquired, including without limitation, method, process, and apparatus claims, in any patent Licensable by grantor. |
|  |  |  |
|  |  | 1.12. "Source Code" means (a) the common form of computer software code in which modifications are made and (b) associated documentation included in or with such code. |
|  |  |  |
|  |  | 1.13. "You" (or "Your") means an individual or a legal entity exercising rights under, and complying with all of the terms of, this License. For legal entities, "You" includes any entity which controls, is controlled by, or is under common control with You. For purposes of this definition, "control" means (a) the power, direct or indirect, to cause the direction or management of such entity, whether by contract or otherwise, or (b) ownership of more than fifty percent (50%) of the outstanding shares or beneficial ownership of such entity. |
|  |  |  |
|  |  | 2. License Grants. |
|  |  |  |
|  |  | 2.1. The Initial Developer Grant. |
|  |  |  |
|  |  | Conditioned upon Your compliance with Section 3.1 below and subject to third party intellectual property claims, the Initial Developer hereby grants You a world-wide, royalty-free, non-exclusive license: |
|  |  |  |
|  |  | (a) under intellectual property rights (other than patent or trademark) Licensable by Initial Developer, to use, reproduce, modify, display, perform, sublicense and distribute the Original Software (or portions thereof), with or without Modifications, and/or as part of a Larger Work; and |
|  |  |  |
|  |  | (b) under Patent Claims infringed by the making, using or selling of Original Software, to make, have made, use, practice, sell, and offer for sale, and/or otherwise dispose of the Original Software (or portions thereof). |
|  |  |  |
|  |  | (c) The licenses granted in Sections 2.1(a) and (b) are effective on the date Initial Developer first distributes or otherwise makes the Original Software available to a third party under the terms of this License. |
|  |  |  |
|  |  | (d) Notwithstanding Section 2.1(b) above, no patent license is granted: (1) for code that You delete from the Original Software, or (2) for infringements caused by: (i) the modification of the Original Software, or (ii) the combination of the Original Software with other software or devices. |
|  |  |  |
|  |  | 2.2. Contributor Grant. |
|  |  |  |
|  |  | Conditioned upon Your compliance with Section 3.1 below and subject to third party intellectual property claims, each Contributor hereby grants You a world-wide, royalty-free, non-exclusive license: |
|  |  |  |
|  |  | (a) under intellectual property rights (other than patent or trademark) Licensable by Contributor to use, reproduce, modify, display, perform, sublicense and distribute the Modifications created by such Contributor (or portions thereof), either on an unmodified basis, with other Modifications, as Covered Software and/or as part of a Larger Work; and |
|  |  |  |
|  |  | (b) under Patent Claims infringed by the making, using, or selling of Modifications made by that Contributor either alone and/or in combination with its Contributor Version (or portions of such combination), to make, use, sell, offer for sale, have made, and/or otherwise dispose of: (1) Modifications made by that Contributor (or portions thereof); and (2) the combination of Modifications made by that Contributor with its Contributor Version (or portions of such combination). |
|  |  |  |
|  |  | (c) The licenses granted in Sections 2.2(a) and 2.2(b) are effective on the date Contributor first distributes or otherwise makes the Modifications available to a third party. |
|  |  |  |
|  |  | (d) Notwithstanding Section 2.2(b) above, no patent license is granted: (1) for any code that Contributor has deleted from the Contributor Version; (2) for infringements caused by: (i) third party modifications of Contributor Version, or (ii) the combination of Modifications made by that Contributor with other software (except as part of the Contributor Version) or other devices; or (3) under Patent Claims infringed by Covered Software in the absence of Modifications made by that Contributor. |
|  |  |  |
|  |  | 3. Distribution Obligations. |
|  |  |  |
|  |  | 3.1. Availability of Source Code. |
|  |  |  |
|  |  | Any Covered Software that You distribute or otherwise make available in Executable form must also be made available in Source Code form and that Source Code form must be distributed only under the terms of this License. You must include a copy of this License with every copy of the Source Code form of the Covered Software You distribute or otherwise make available. You must inform recipients of any such Covered Software in Executable form as to how they can obtain such Covered Software in Source Code form in a reasonable manner on or through a medium customarily used for software exchange. |
|  |  |  |
|  |  | 3.2. Modifications. |
|  |  |  |
|  |  | The Modifications that You create or to which You contribute are governed by the terms of this License. You represent that You believe Your Modifications are Your original creation(s) and/or You have sufficient rights to grant the rights conveyed by this License. |
|  |  |  |
|  |  | 3.3. Required Notices. |
|  |  |  |
|  |  | You must include a notice in each of Your Modifications that identifies You as the Contributor of the Modification. You may not remove or alter any copyright, patent or trademark notices contained within the Covered Software, or any notices of licensing or any descriptive text giving attribution to any Contributor or the Initial Developer. |
|  |  |  |
|  |  | 3.4. Application of Additional Terms. |
|  |  |  |
|  |  | You may not offer or impose any terms on any Covered Software in Source Code form that alters or restricts the applicable version of this License or the recipients rights hereunder. You may choose to offer, and to charge a fee for, warranty, support, indemnity or liability obligations to one or more recipients of Covered Software. However, you may do so only on Your own behalf, and not on behalf of the Initial Developer or any Contributor. You must make it absolutely clear that any such warranty, support, indemnity or liability obligation is offered by You alone, and You hereby agree to indemnify the Initial Developer and every Contributor for any liability incurred by the Initial Developer or such Contributor as a result of warranty, support, indemnity or liability terms You offer. |
|  |  |  |
|  |  | 3.5. Distribution of Executable Versions. |
|  |  |  |
|  |  | You may distribute the Executable form of the Covered Software under the terms of this License or under the terms of a license of Your choice, which may contain terms different from this License, provided that You are in compliance with the terms of this License and that the license for the Executable form does not attempt to limit or alter the recipient's rights in the Source Code form from the rights set forth in this License. If You distribute the Covered Software in Executable form under a different license, You must make it absolutely clear that any terms which differ from this License are offered by You alone, not by the Initial Developer or Contributor. You hereby agree to indemnify the Initial Developer and every Contributor for any liability incurred by the Initial Developer or such Contributor as a result of any such terms You offer. |
|  |  |  |
|  |  | 3.6. Larger Works. |
|  |  |  |
|  |  | You may create a Larger Work by combining Covered Software with other code not governed by the terms of this License and distribute the Larger Work as a single product. In such a case, You must make sure the requirements of this License are fulfilled for the Covered Software. |
|  |  |  |
|  |  | 4. Versions of the License. |
|  |  |  |
|  |  | 4.1. New Versions. |
|  |  |  |
|  |  | Sun Microsystems, Inc. is the initial license steward and may publish revised and/or new versions of this License from time to time. Each version will be given a distinguishing version number. Except as provided in Section 4.3, no one other than the license steward has the right to modify this License. |
|  |  |  |
|  |  | 4.2. Effect of New Versions. |
|  |  |  |
|  |  | You may always continue to use, distribute or otherwise make the Covered Software available under the terms of the version of the License under which You originally received the Covered Software. If the Initial Developer includes a notice in the Original Software prohibiting it from being distributed or otherwise made available under any subsequent version of the License, You must distribute and make the Covered Software available under the terms of the version of the License under which You originally received the Covered Software. Otherwise, You may also choose to use, distribute or otherwise make the Covered Software available under the terms of any subsequent version of the License published by the license steward. |
|  |  |  |
|  |  | 4.3. Modified Versions. |
|  |  |  |
|  |  | When You are an Initial Developer and You want to create a new license for Your Original Software, You may create and use a modified version of this License if You: (a) rename the license and remove any references to the name of the license steward (except to note that the license differs from this License); and (b) otherwise make it clear that the license contains terms which differ from this License. |
|  |  |  |
|  |  | 5. DISCLAIMER OF WARRANTY. |
|  |  |  |
|  |  | COVERED SOFTWARE IS PROVIDED UNDER THIS LICENSE ON AN "AS IS" BASIS, WITHOUT WARRANTY OF ANY KIND, EITHER EXPRESSED OR IMPLIED, INCLUDING, WITHOUT LIMITATION, WARRANTIES THAT THE COVERED SOFTWARE IS FREE OF DEFECTS, MERCHANTABLE, FIT FOR A PARTICULAR PURPOSE OR NON-INFRINGING. THE ENTIRE RISK AS TO THE QUALITY AND PERFORMANCE OF THE COVERED SOFTWARE IS WITH YOU. SHOULD ANY COVERED SOFTWARE PROVE DEFECTIVE IN ANY RESPECT, YOU (NOT THE INITIAL DEVELOPER OR ANY OTHER CONTRIBUTOR) ASSUME THE COST OF ANY NECESSARY SERVICING, REPAIR OR CORRECTION. THIS DISCLAIMER OF WARRANTY CONSTITUTES AN ESSENTIAL PART OF THIS LICENSE. NO USE OF ANY COVERED SOFTWARE IS AUTHORIZED HEREUNDER EXCEPT UNDER THIS DISCLAIMER. |
|  |  |  |
|  |  | 6. TERMINATION. |
|  |  |  |
|  |  | 6.1. This License and the rights granted hereunder will terminate automatically if You fail to comply with terms herein and fail to cure such breach within 30 days of becoming aware of the breach. Provisions which, by their nature, must remain in effect beyond the termination of this License shall survive. |
|  |  |  |
|  |  | 6.2. If You assert a patent infringement claim (excluding declaratory judgment actions) against Initial Developer or a Contributor (the Initial Developer or Contributor against whom You assert such claim is referred to as "Participant") alleging that the Participant Software (meaning the Contributor Version where the Participant is a Contributor or the Original Software where the Participant is the Initial Developer) directly or indirectly infringes any patent, then any and all rights granted directly or indirectly to You by such Participant, the Initial Developer (if the Initial Developer is not the Participant) and all Contributors under Sections 2.1 and/or 2.2 of this License shall, upon 60 days notice from Participant terminate prospectively and automatically at the expiration of such 60 day notice period, unless if within such 60 day period You withdraw Your claim with respect to the Participant Software against such Participant either unilaterally or pursuant to a written agreement with Participant. |
|  |  |  |
|  |  | 6.3. In the event of termination under Sections 6.1 or 6.2 above, all end user licenses that have been validly granted by You or any distributor hereunder prior to termination (excluding licenses granted to You by any distributor) shall survive termination. |
|  |  |  |
|  |  | 7. LIMITATION OF LIABILITY. |
|  |  |  |
|  |  | UNDER NO CIRCUMSTANCES AND UNDER NO LEGAL THEORY, WHETHER TORT (INCLUDING NEGLIGENCE), CONTRACT, OR OTHERWISE, SHALL YOU, THE INITIAL DEVELOPER, ANY OTHER CONTRIBUTOR, OR ANY DISTRIBUTOR OF COVERED SOFTWARE, OR ANY SUPPLIER OF ANY OF SUCH PARTIES, BE LIABLE TO ANY PERSON FOR ANY INDIRECT, SPECIAL, INCIDENTAL, OR CONSEQUENTIAL DAMAGES OF ANY CHARACTER INCLUDING, WITHOUT LIMITATION, DAMAGES FOR LOST PROFITS, LOSS OF GOODWILL, WORK STOPPAGE, COMPUTER FAILURE OR MALFUNCTION, OR ANY AND ALL OTHER COMMERCIAL DAMAGES OR LOSSES, EVEN IF SUCH PARTY SHALL HAVE BEEN INFORMED OF THE POSSIBILITY OF SUCH DAMAGES. THIS LIMITATION OF LIABILITY SHALL NOT APPLY TO LIABILITY FOR DEATH OR PERSONAL INJURY RESULTING FROM SUCH PARTY'S NEGLIGENCE TO THE EXTENT APPLICABLE LAW PROHIBITS SUCH LIMITATION. SOME JURISDICTIONS DO NOT ALLOW THE EXCLUSION OR LIMITATION OF INCIDENTAL OR CONSEQUENTIAL DAMAGES, SO THIS EXCLUSION AND LIMITATION MAY NOT APPLY TO YOU. |
|  |  |  |
|  |  | 8. U.S. GOVERNMENT END USERS. |
|  |  |  |
|  |  | The Covered Software is a "commercial item," as that term is defined in 48 C.F.R. 2.101 (Oct. 1995), consisting of "commercial computer software" (as that term is defined at 48 C.F.R. 252.227-7014(a)(1)) and "commercial computer software documentation" as such terms are used in 48 C.F.R. 12.212 (Sept. 1995). Consistent with 48 C.F.R. 12.212 and 48 C.F.R. 227.7202-1 through 227.7202-4 (June 1995), all U.S. Government End Users acquire Covered Software with only those rights set forth herein. This U.S. Government Rights clause is in lieu of, and supersedes, any other FAR, DFAR, or other clause or provision that addresses Government rights in computer software under this License. |
|  |  |  |
|  |  | 9. MISCELLANEOUS. |
|  |  |  |
|  |  | This License represents the complete agreement concerning subject matter hereof. If any provision of this License is held to be unenforceable, such provision shall be reformed only to the extent necessary to make it enforceable. This License shall be governed by the law of the jurisdiction specified in a notice contained within the Original Software (except to the extent applicable law, if any, provides otherwise), excluding such jurisdiction's conflict-of-law provisions. Any litigation relating to this License shall be subject to the jurisdiction of the courts located in the jurisdiction and venue specified in a notice contained within the Original Software, with the losing party responsible for costs, including, without limitation, court costs and reasonable attorneys' fees and expenses. The application of the United Nations Convention on Contracts for the International Sale of Goods is expressly excluded. Any law or regulation which provides that the language of a contract shall be construed against the drafter shall not apply to this License. You agree that You alone are responsible for compliance with the United States export administration regulations (and the export control laws and regulation of any other countries) when You use, distribute or otherwise make available any Covered Software. |
|  |  |  |
|  |  | 10. RESPONSIBILITY FOR CLAIMS. |
|  |  |  |
|  |  | As between Initial Developer and the Contributors, each party is responsible for claims and damages arising, directly or indirectly, out of its utilization of rights under this License and You agree to work with Initial Developer and Contributors to distribute such responsibility on an equitable basis. Nothing herein is intended or shall be deemed to constitute any admission of liability. |
|  |  |  |

******** End of Document ********
