# Supplementary material for: Data based predictive models for odor perception
Source: Sci Rep. 2020 Oct 13;10:17136. doi: 10.1038/s41598-020-73978-1 (PMC7553929; doi:10.1038/s41598-020-73978-1)
Supplement: Supplementary file 3 — Supplementary file3 [file 41598_2020_73978_MOESM3_ESM.zip › .svn/text-base/READ ME_Vitellus V 1.2.1.docx.svn-base]

# READ ME

**TCS LICENSED PROGRAM: Vitellus**

**VERSION: 1.2.1**

The below table shows the list of open source components included in the Licensed Program as mentioned in the Notices file and their location in the source code.

| **NON TCS File/Components/libraries etc.** | | | |
| --- | --- | --- | --- |
| **File/Folder** | **Component** | **License** | **Usage** |
| WebContent/WEB-INF/lib/commons-collections-3.2.1.jar | Apache Commons Collections | Apache License 2.0 | File |
| WebContent/WEB-INF/lib/log4j-1.2.12.jar | Apache Log4j | Apache License 2.0 | File |
| WebContent/WEB-INF/lib/httpcore-4.2.5.jar | Apache Nutch | Apache License 2.0 | File |
| WebContent/WEB-INF/lib/commons-io-2.4.jar | Apache Rave | Apache License 2.0 | File |
| WebContent/WEB-INF/lib/scala-compiler.jar, WebContent/WEB-INF/lib/scala-library.jar | Apache Spark | Apache License 2.0 | File |
| WebContent/WEB-INF/lib/xmlbeans-2.3.0.jar | Apache XMLBeans | Apache License 2.0 | File |
| WebContent/WEB-INF/lib/commons-beanutils-1.8.0.jar | Apache-Jakarta BeanUtils | Apache License 2.0 | File |
| WebContent/WEB-INF/lib/commons-codec-1.4.jar | Apache-Jakarta Codec | Apache License 2.0 | File |
| WebContent/WEB-INF/lib/commons-codec-1.4.jar | bizmoso | Apache License 2.0 | File |
| WebContent/themes/showcase/images/ui-icons_f5e175_256x240.png | BlueOxygen Cimande | Apache License 2.0 | File |
| WebContent/js/zoom.js | bubble-map | MIT License | Snippet |
| WebContent/js/d3.js | c3-angular-sample | MIT License | File |
| WebContent/js/ext/splitter.js | codebox7 | Apache License 2.0 | Snippet (+ File) |
| WebContent/WEB-INF/lib/commons-lang3-3.4.jar | commons-lang3-3.4.jar | Apache License 2.0 | Component (Dynamic Library) |
| WebContent/Report_Download/Template/js/jquery.min.js | genie-web | Apache License 2.0 | File |
| WebContent/WEB-INF/lib/jcl-over-slf4j-1.5.8.jar | grails-spring-security-cas | Apache License 2.0 | File |
| WebContent/WEB-INF/lib/hive-common.jar | hive-common.jar | Apache License 2.0 | Component (Dynamic Library) |
| WebContent/WEB-INF/lib/quartz-1.6.2.jar | j2se-codeserver | BSD 3-clause "New" or "Revised" License | File |
| WebContent/fonts/glyphicons-halflings-regular.woff2 | JBPM Form Modeler Showcase WebApp | Apache License 2.0 | Snippet (+ File) |
| WebContent/css/images/ajax-loader1.gif | Joinfuse | MIT License | File |
| WebContent/js/jquery-ui.js | jQuery UI (Combined Library) | MIT License | File |
| WebContent/css/asc.gif | jQuery UI - jquery-ui from code.google.com | MIT License | File |
| WebContent/js/ext/jquery.msgBox.js | jquery.msgBox.js | MIT License | File |
| WebContent/js/ext/jquery.pnotify.min_backup.js | jQuery4PHP | MIT License | File |
| WebContent/js/ext/jquery.validationEngine-en.js | jsdelivr | MIT License | Snippet (+ File) |
| WebContent/js/colResizable-1.5.min.js | lordofgrid | ISC License | File |
| WebContent/WEB-INF/lib/mysql-connector-java-5.1.18.jar | Mifos- MicroFinance Open Source | Apache License 2.0 | File |
| WebContent/js/moment.js | moment | MIT License | File |
| WebContent/css/ext/images/msgBoxBackGround.png | msgBoxBackGround.png | MIT License | File |
| WebContent/js/razorflow.devtools.min.js | nonglam-fashionshop-dh10dt | Apache License 2.0 | Snippet |
| WebContent/js/ext/jquery-ui-1.8.19.custom.min.js | PFTT2 | BSD 3-clause "New" or "Revised" License | File |
| WebContent/js/zoom.js | plotly.js | MIT License | Snippet |
| WebContent/WEB-INF/jsp/homePage.jsp | proyecto6tosvnp | Apache License 2.0 | Snippet |
| WebContent/js/d3.parsets.js | rails-data-explorer | MIT License | Snippet |
| WebContent/js/underscore-min.js | Sail.JavaScript | Apache License 2.0 | Snippet |
| WebContent/WEB-INF/lib/scalaj-collection_2.10-1.6.jar | scalaj-collection_2.10-1.6.jar | Apache License 2.0 | Component (Dynamic Library) |
| WebContent/WEB-INF/lib/hive-jdbc.jar | sequel-impala | MIT License | File |
| WebContent/WEB-INF/lib/slf4j-api-1.7.12.jar | Simple Logging Facade for Java (SLF4J) | MIT License | File |
| WebContent/WEB-INF/lib/commons-csv-1.1.jar | The Open For Business Project | MIT License | File |
| WebContent/js/topojson.v1.min.js | topojson | BSD 3-clause "New" or "Revised" License | File |
| WebContent/WEB-INF/lib/javax.el-api-2.2.4.jar | vraptor | Apache License 2.0 | File |
| WebContent/js/ext/jquery.dynatree.min.js | webgme | MIT License | File |
| .classpath | wson-application-wsroot | Apache License 2.0 | File |
| WebContent/Report_Download/Template/js/x3dom-full.js , WebContent/js/x3dom-full.js | x3dom | MIT License | Snippet |
| WebContent/js/ext/jquery.filedownload.js | TireBias | Microsoft Public License | Snippet |
